# Controllable synthesis and structural design of novel all-organic polymers toward high energy storage dielectrics

**DOI:** 10.3389/fchem.2022.979926

**Published:** 2022-08-17

**Authors:** Honghong Gong, Qinglong Ji, Yipin Cheng, Jie Xiong, Meirong Zhang, Zhicheng Zhang

**Affiliations:** ^1^ Xi’an Key Laboratory of Sustainable Energy Materials Chemistry, Department of Applied Chemistry, School of Chemistry, Xi’an Jiaotong University, Xi’an, Shaanxi, China; ^2^ Xi’an Jiaotong University Suzhou Academy, Suzhou, Jiangsu, China

**Keywords:** dielectric capacitors, all-organic polymer dielectric, controlled/living radical polymerization, dipole regulation, multilayer structure

## Abstract

As the core unit of energy storage equipment, high voltage pulse capacitor plays an indispensable role in the field of electric power system and electromagnetic energy related equipment. The mostly utilized polymer materials are metallized polymer thin films, which are represented by biaxially oriented polypropylene (BOPP) films, possessing the advantages including low cost, high breakdown strength, excellent processing ability, and self-healing performance. However, the low dielectric constant (*ε*_r_ < 3) of traditional BOPP films makes it impossible to meet the demand for increased high energy density. Controlled/living radical polymerization (CRP) and related techniques have become a powerful approach to tailor the chemical and physical properties of materials and have given rise to great advances in tuning the properties of polymer dielectrics. Although organic-inorganic composite dielectrics have received much attention in previous studies, all-organic polymer dielectrics have been proven to be the most promising choice because of its light weight and easy large-scale continuous processing. In this short review, we begin with some basic theory of polymer dielectrics and some theoretical considerations for the rational design of dielectric polymers with high performance. In the guidance of these theoretical considerations, we review recent progress toward all-organic polymer dielectrics based on two major approaches, one is to control the polymer chain structure, containing microscopic main-chain and side-chain structures, by the method of CRP and the other is macroscopic structure design of all-organic polymer dielectric films. And various chemistry and compositions are discussed within each approach.

## Introduction

**FIGURE 1:**
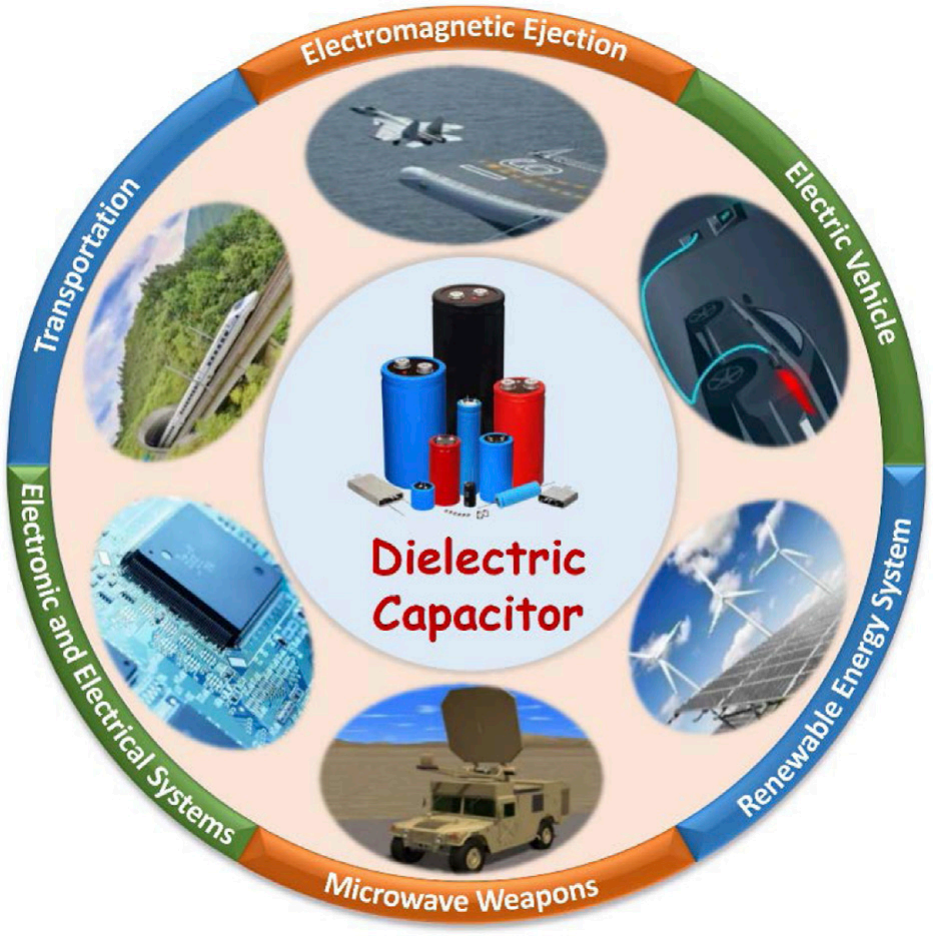
Application fields of dielectric capacitors, including electric vehicle, transportation, electronic and electrical systems, renewable energy system, electromagnetic ejection, and microwave weapons.

High-voltage pulse capacitors are widely used in electronic and electrical systems, optoelectronic and electromagnetic equipment due to their ultra-fast charging and discharging capabilities, ultra-high-power density, and excellent AC and DC high-voltage characteristics. In recent years, with the rapid development of industries such as electric vehicles, high-power microwave and laser systems, and electromagnetic launch and ejection systems, the demand for metallized film capacitors with high *U*_e_ has increased significantly (Figure 1). As one of the most important parts of high pulse film capacitors, dielectric materials with high *U*_e_ and low dielectric loss exhibit huge value and demands in the national economy and national defense construction (Huan et al., 2016; Qiao et al., 2018; Fan et al., 2019). The existing high-voltage pulse capacitors are mainly made of ceramic materials, which have the advantages of excellent temperature characteristics and AC and DC high-voltage characteristics. However, the density of ceramic materials is high and the *E*_b_ is low (<100 MV/m), so the thickness of ceramic between electrodes is high when applied at high voltage, resulting in bulky capacitors. In addition, the sintering process of ceramics makes it difficult for large-area thin film fabrication and flexibility (Prateek et al., 2016; Kang et al., 2018; Shen et al., 2018; Yang et al., 2019). Compared with ceramic dielectric materials, all-organic polymer dielectric materials have overwhelming advantages in large-area thin film fabrication, capacitor rolling, and capacitor *U*_e_. The currently used all-organic film capacitors based on PET, PP, PS, PPS, PC, etc. generally have the shortcoming of low *U*_e_ (Nasreen et al., 2018; Li Q. et al., 2019; Li et al., 2019b; Li et al., 2019c; Li et al., 2021a; Chunarrom and Manuspiya, 2021). Taking the most widely used BOPP film as an example, it has the advantages of low dielectric loss, high *E*_b_, easy film preparation and high cost performance. However, due to the small *ε*_r_ (≈2.2), the *U*_e_ is only 2.5–3.0 J/cm^3^ at a high electric field of 600 MV/m. This is far from meeting the design requirements of advanced electronic power equipment for miniaturization or even miniaturization of energy storage devices, so it is imminent to greatly improve the energy storage density of dielectrics.

**FIGURE 2:**
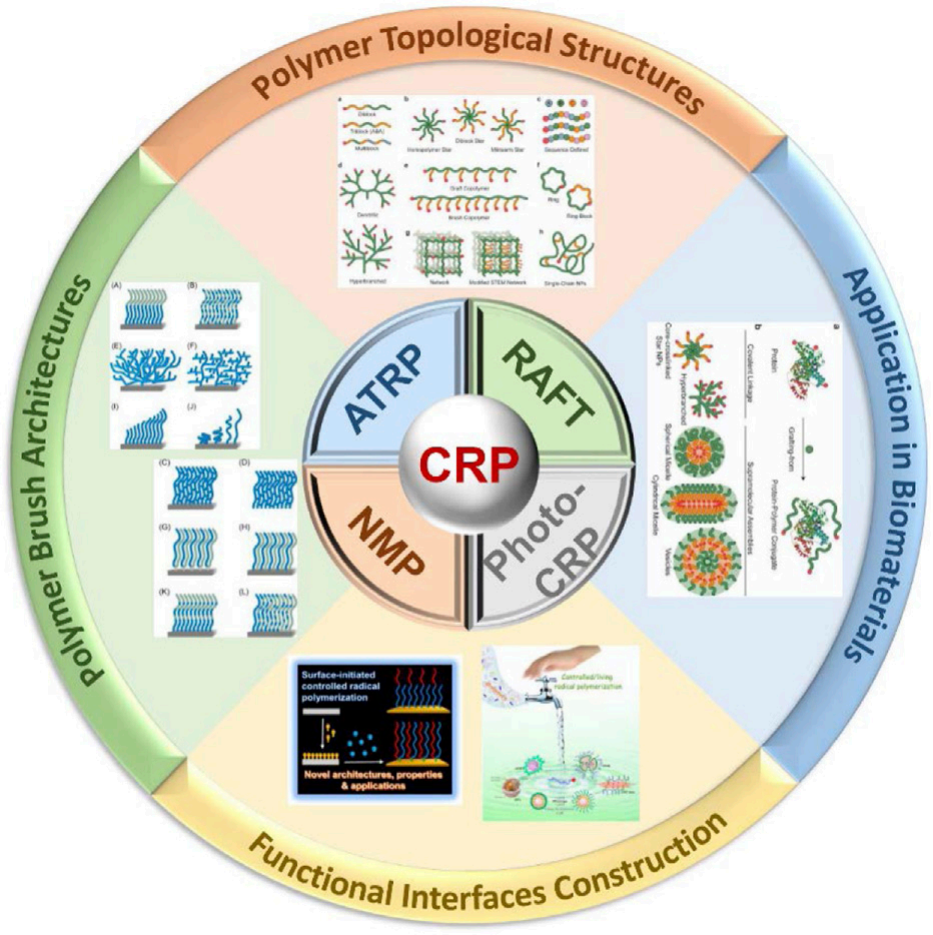
Classification of typical CRPs and different topologies of polymers synthesized by CRP technology.

Controlled/living radical polymerization (CRP), such as ATRP, RAFT polymerization, NMP and related techniques, have transformed materials synthesis over the past two decades by providing polymer chemists with powerful tools that enable control over architecture, composition and chain length distributions (Zhou et al., 2022a; Dau et al., 2022; Fromel et al., 2022; Qiao et al., 2022). Polymers with various topologies, such as block, graft, gradient, star, brush, dendrimer and hyperbranched polymers could be synthesized by CRP technology. In addition, CRP is also a commonly used method to modify materials in order to adjust their chemical and physical properties (Figure 2) (Bagheri et al., 2021; Corrigan et al., 2021; Gong et al., 2021; Zhou et al., 2021; Antonopoulou et al., 2022; Zhang et al., 2022b; Li et al., 2022b; Zhou et al., 2022c; Corbin and Miyake, 2022; Dworakowska et al., 2022). ROMP is a relatively new research field in the preparation of polymer materials, which has all the characteristics of olefin metathesis reactions. Due to its high reactivity, it has become a widely used synthetic method for the preparation of polymer materials with controllable structures (Lu L. et al., 2020; Varlas et al., 2020; Huang et al., 2021; Blosch et al., 2022a; Feist et al., 2022; Quach et al., 2022; Yu et al., 2022). In the past few decades, most of the research on ROMP has focused on its reaction mechanism, catalysts, and the exploration of new monomers. In recent years, polymer materials with specific structures and properties have attracted more and more attention with the development of science and technology. The precise preparation of functional polymers has high requirements on the synthetic method used. From the perspective of chemistry and materials, ROMP, as a polymerization method with controllable activity, fast reaction speed and high efficiency, has become a very powerful and versatile approach for constructing functional polymer materials with different topological structures like CRP (Barbey et al., 2009; Jennings et al., 2016; Wang et al., 2016; Zoppe et al., 2017; Corrigan et al., 2020; Messina et al., 2020; Blosch et al., 2022b).

From the calculation formula of *U*_e_ = 1/2*ε*_0_ *ε*_r_ *E*_b_^2^, it can be seen that there are two main ways to obtain high *U*_e_ in dielectrics, namely, increasing *ε*_r_ and *E*_b_. Therefore, in recent years, domestic and foreign researchers have made a lot of useful attempts to improve *ε*_r_ and *E*_b_ of dielectrics. The composites obtained by adding high *ε*_r_ inorganic fillers into the polymer matrix can significantly improve the *ε*_r_ of the polymer dielectrics. However, due to the huge electrical mismatch between the polymer matrix and the ceramic filler, there will inevitably be defects near the filler-matrix interface inside the composite (Prateek et al., 2016; Huang et al., 2019; Li Q. et al., 2021; Hu et al., 2021; Ren et al., 2021; Zhang X. et al., 2022). These defects will become breakdown weak points under an applied electric field, which will greatly reduce the *E*_b_ of the composite material. In addition, due to the existence of interface defects, it will lead to a large dielectric loss, thus reducing the *η*. It seems to be a good option that increase *ε*_r_ of existing polymer dielectrics to rise their *U*_e_. Chung TC et al. introduced a certain number of polar groups (such as -OH, -NH_2_, etc.) into PP, because the polar groups can contribute to a higher induced electron polarization, which improves the polarization strength, and the *ε*_r_ of the obtained BOPP film can be increased to 4. However, the introduction of polar groups disrupts the isotacticity of the original molecular chain, resulting in a decrease in the crystallinity of the polymer, and with the increase of the introduced polar groups, the energy loss inevitably rises rapidly. What’s more, the introduction of polar groups in PP requires tedious operations such as harsh protection and deprotection of polar groups under anhydrous and oxygen-free conditions, and the practicability of the process is very poor (Yuan et al., 2010).

In recent years, researchers have paid more and more attention to amorphous glassy polymer dielectrics. There is no difference between the crystalline phase and the amorphous phase of glassy polymer, so the introduction of polar groups will not cause changes in crystalline properties. More importantly, many glassy polymers have high *T*_g_s and are expected to be used in high temperature dielectrics (Wu et al., 2013; Zhu et al., 2018; Li et al., 2019d). For example, Zhu et al., 2018 prepared a glassy polyurea containing aromatic rings in the main chain, and the *U*_e_ under optimal conditions reaches 12.0 J/cm^3^, which is nearly 4 times that of BOPP. However, for this glassy polymer, while increasing the *ε*_r_ through the interaction between dipoles, the high dielectric loss caused by the relaxation of the dipole polarization is also unavoidable (Treufeld et al., 2014).

Thus far, various effective strategies have been developed to improve the inherent low *U*_e_ of polymer dielectrics. However, enhanced *U*_e_ is always accompanied by suppressed *η*, which is detrimental to practical applications and deserves considerable concern. More recently, the polymer dielectrics with optimized hierarchically layered structures has become an emerging approach to resolve the existing paradox between high *ε*_r_ and high breakdown strength in single-layered dielectric films, which resulted in substantial improvement in their capacitive energy storage performance. It is demonstrated that the electric field distribution, breakdown strength and capacitive performance can be readily adjusted by systematically varying the interfaces, chemical structures and ratios of the constituent layers (Qiao et al., 2018; Hu et al., 2020; Wei and Zhu, 2020; Feng M. et al., 2021; Feng et al., 2022; Wu et al., 2022; Yang et al., 2022).

Based on the above-mentioned information, this review summarizes the research advances of all-organic polymer dielectric, including controllable synthesis using CRP or ROMP method and structural design such as introducing hydrogen bonds and all-organic layered films in the field of high-energy-density capacitors. The efficient strategies for the preparation of all-organic polymer dielectric have been highlighted.

## Basic theories on dielectric for energy storage

### Principle of energy storage capacitor

**FIGURE 3:**
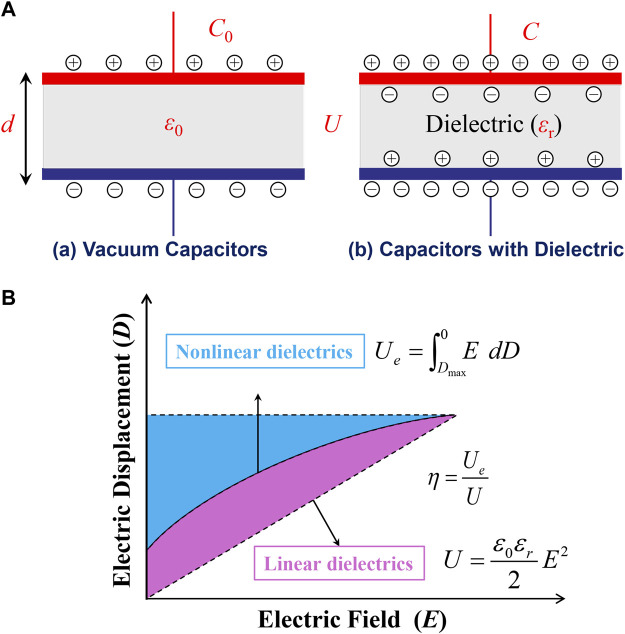
**(A)** The principle of capacitor energy storage; **(B)** Schematic *D*−*E* hysteresis loop used for the calculation of energy density.

Capacitors are passive electronic components that can store electrostatic charges (Feng et al., 2022). The most basic structure consists of two parallel metal plates, namely positive and negative electrodes, and an intermediate insulating material, namely dielectric. As shown in Figure 3A, when there is no dielectric material (vacuum), the capacitor is charged with a constant voltage *U*, the charge on the plate is *Q*_0_, and the electric field strength between the two electrode plates is *E*_0_. When the dielectric material is filled between the two electrodes, the dielectric is polarized under the action of the electric field. The bound charges (electrons, ions, dipoles, etc.) inside the dielectric material undergo limited short-range migration under the action of the electric field to form induced dipoles; a large number of induced dipoles are connected end to end, and finally induced charges are generated on the surface of the dielectric. Because the polarization electric field generated by the medium is opposite to the direction of the external electric field, it will cancel out part of the effect of the external electric field. The result is that at a given voltage *U*, more charge is induced to be stored in the electrode plates. That is, after introducing the medium of relative permittivity *ε*_r_, the capacitance *C* becomes:

$$C=\frac{\varepsilon_{r}\varepsilon_{0}S}{d}$$ (1)

where *ε*_0_ is the vacuum dielectric constant (8.85 × 10^−12^ F/m), *S* is the area of the electrode plate, and *d* is the distance between the electrode plates, that is, the thickness of the dielectric material. The polarization of the dielectric material reduces the electric field strength between the plate capacitors to 1/*ε*_r_ in vacuum and increases the capacitance by a factor of *ε*_r_. Therefore, the *ε*_r_ of the dielectric material characterizes the ability of the dielectric material to generate a polarization electric field under the condition of a set polarization voltage.

In addition, the dielectric material consumes thermal energy due to the relaxation and heating of the dipole moment under the alternating electric field, resulting in dielectric loss. At this time, the dielectric constant is rewritten in complex form:

ε=ε′−iε″ (2)

The tangent of the dielectric loss angle is used to describe the degree of dielectric loss,

$$\text{tan}\delta =\frac{\varepsilon \prime \prime }{\varepsilon \prime }$$ (3)

Because the charge bound on the surface of the polarized dielectric material cancels out part of electric field from free charge on the electrodes, the electric field strength actually suffered by the dielectric material decreases after the introduction of the dielectric material between the electrodes. And the difference between them represents depolarization electric field strength. Introducing the concepts of charge displacement *D*, polarization strength *P* and polarizability *α*, when there is a dielectric material, under external electric field *E*, there are:

$$D=\varepsilon_{0}E+P=\varepsilon_{0}E+\alpha \varepsilon_{0}E=\varepsilon_{r}\varepsilon_{0}E$$ (4)

For linear dielectrics, the energy density of per unit volume *U*_e_ is (shown in Figure 3B):

$$U_{e}=\frac{W}{V}=\frac{1}{2}\varepsilon_{r}\varepsilon_{0}E^{2}$$ (5)

It can be seen from Eq. 5 that the *U*_e_ of capacitor depends on the *ε*_r_ of the dielectric material and the working electric field strength *E*_b_.

What’s more, part of the charging energy inevitably cannot be released due to dielectric loss during the charge-discharge process of dielectric. Therefore, the *η* of the dielectric capacitor is determined by the charged energy *U* and discharge energy *U*_e_ as follows:

$$\eta =\frac{U_{e}}{U}$$ (6)

### Dielectric polarization mechanism

According to the different polarization mechanisms, the polarization process of dielectrics can be divided into electronic polarization, ionic polarization, orientation polarization and interface polarization (Prateek et al., 2016; Feng et al., 2022). The same dielectric may involve several different electrical polarization mechanisms. At the same time, each electric polarization mechanism has its main active frequency and its characteristic cut-off frequency. If the cut-off frequency is exceeded, the corresponding mechanism cannot vibrate with the electromagnetic wave and can no longer contribute to the electric polarization. For each dielectric material, the cut-off frequency of the electrical polarization mechanism and the degree of electrical polarization are also different.

Electronic polarization refers to the phenomenon that the electron cloud outside each nucleus in the molecule moves relative to the nucleus under the external electric field, which makes the positive and negative charges center of the molecule change. Due to the large binding force of the electrons in the inner layer of the atom, the displacement is not easy to occur, and the electron displacement polarization only occurs in the outer valence electrons. The electron polarization process is elastically reversible and does not consume energy. In addition, the time required for the electron polarization process is extremely short (only about 10^−15^–10^−13^ s) due to the high speed of electron movement. In the ultraviolet frequency domain, the electron polarization no longer responds.

Ionic polarization refers to the phenomenon that the atoms connected to the polymer backbone undergo relative displacement under an external electric field, resulting in the change of the center of the molecular charge. Usually, the atoms in the molecule have more or less positive or negative apparent charges due to bonding, and a certain degree of offset occurs under an external electric field. The atomic polarization is generally quite small, only 1/10 of the electron polarization, and due to the large atomic mass and long response time, the atomic polarization time required is more than 10^−13^ s. In the infrared or far-infrared frequency domain, atomic polarization loses its ability to respond to external electric fields. Electron polarization and atomic polarization are both caused by the displacement of the centers of positive and negative charges in molecules under an external electric field, also known as induced displacement polarization.

Orientation polarization refers to the phenomenon that the dipoles existing in the polymer material rotate and align along the direction of the electric field under the action of the electric field, resulting in a macroscopic dipole moment. Orientation polarization mainly occurs in polar dielectric materials containing permanent dipoles, whose permanent dipole moments can exist in the backbone of the polymer, or can be attached to the main chain as a functional group on the side chain. Since the rotation of polar molecules along the direction of the external electric field needs to overcome their own inertia and rotational resistance, it takes much longer time to complete the orientation polarization process than displacement polarization, about 10^−2^–10^−10^ s.

Interface polarization refers to a dielectric in an electric field, and the charge carriers inside it may migrate for a certain distance. If the migration of charge carriers is hindered, for example, at the structural interface of a heterogeneous material, the charge accumulation occurs and the interface polarization phenomenon is formed, also known as the Maxwell-Wagner effect. Interface polarization is also known as space charge polarization. The response time of this polarization process is much longer, from seconds to hours or even years (like electrets). In the practical polymer-based dielectrics, the magnitude of interfacial polarization is determined by various factors including the impurities or defects inside materials, crystal−amorphous interfaces, as well as the interface between film and electrode. From an energy storage perspective, the higher the polarization of the dielectric material, the higher the corresponding charge storage density. At the same time, it is required that the charges stored in the dielectric polarization process must be able to be released quickly and reversibly.

## Controllable synthesis of all-organic polymer dielectric

### Application of controlled/living radical polymerization in modification of high-*k* fluoro-polymer dielectric

Fluoropolymers take the advantage of inertness to chemical corrosion, prominent weather resistance, low flammability, thermal stability, and easy processing. They are widely used in microelectronics, optics, aerospace, transducers, etc. (Ameduri, 2018; Wehbi et al., 2020; Lv and Cheng, 2021). Therefore, numerous fluorine-containing polymers have been prepared to fulfill the requirements of versatile applications. Among them, PVDF and its copolymers are the most studied class of materials. PVDF based fluoropolymers have attracted continuously increasing interests as dielectric materials for their relatively high *ε*_r_ of 10–12 besides of their well-known excellent ferro- and piezo-electric properties (Li et al., 2019e; Lu P. et al., 2020; Li R. et al., 2022). However, PVDF based normal ferroelectric polymers render a rather small *U*_e_ owing to the large remnant polarization and low saturation electric field. To release the energy stored, many efforts have been devoted to modify PVDF and its copolymers physically and chemically, and they have been successfully turned from normal ferroelectrics into relaxed ferroelectrics. One of the commonly used chemical methods is “graft-from” ATRP as C-Cl bond on CTFE units as macro-initiator, which can introduce different kinds of side chains to form micro-insulator layers surrounding the ferroelectric domains. We have made a detailed summary of the research in this area before (Gong et al., 2021), and here is only a brief introduction to the application of CRP in the modification of P(VDF-CTFE) or its copolymer.

In 2006, Zhang and Russell (2006) firstly reported that the secondary chlorines in CTFE units of fluoropolymers could able to initiate ATRP of various monomers. After that more and more research groups used this method to modify some important commercial fluoropolymers containing CTFE units, such as P(VDF-CTFE) or P(VDF-CTFE-TrFE). Lei Zhu et al. studied the confined ferroelectric properties in a series of P(VDF-CTFE)-*g*-PS graft copolymers, and their application as high energy density capacitor films. Due to the low polarizability of the confining PS-rich layer at the amorphous-crystalline interface, the compensation polarization is substantially decreased resulting in a novel confined ferroelectric behavior in these graft copolymers (Guan et al., 2011a). Subsequently, the same group reported an antiferroelectric-like polymer P(VDF-TrFE-CTFE)-*g*-PS that was successfully synthesized through macro-initiated ATRP. A significantly reduced ferroelectric loss and relatively high discharged energy density were observed in the P(VDF-TrFE-CTFE)-*g*-PS(14%) graft copolymer because of this antiferroelectric-like behavior even at high poling fields (Guan et al., 2011b).

**FIGURE 4:**
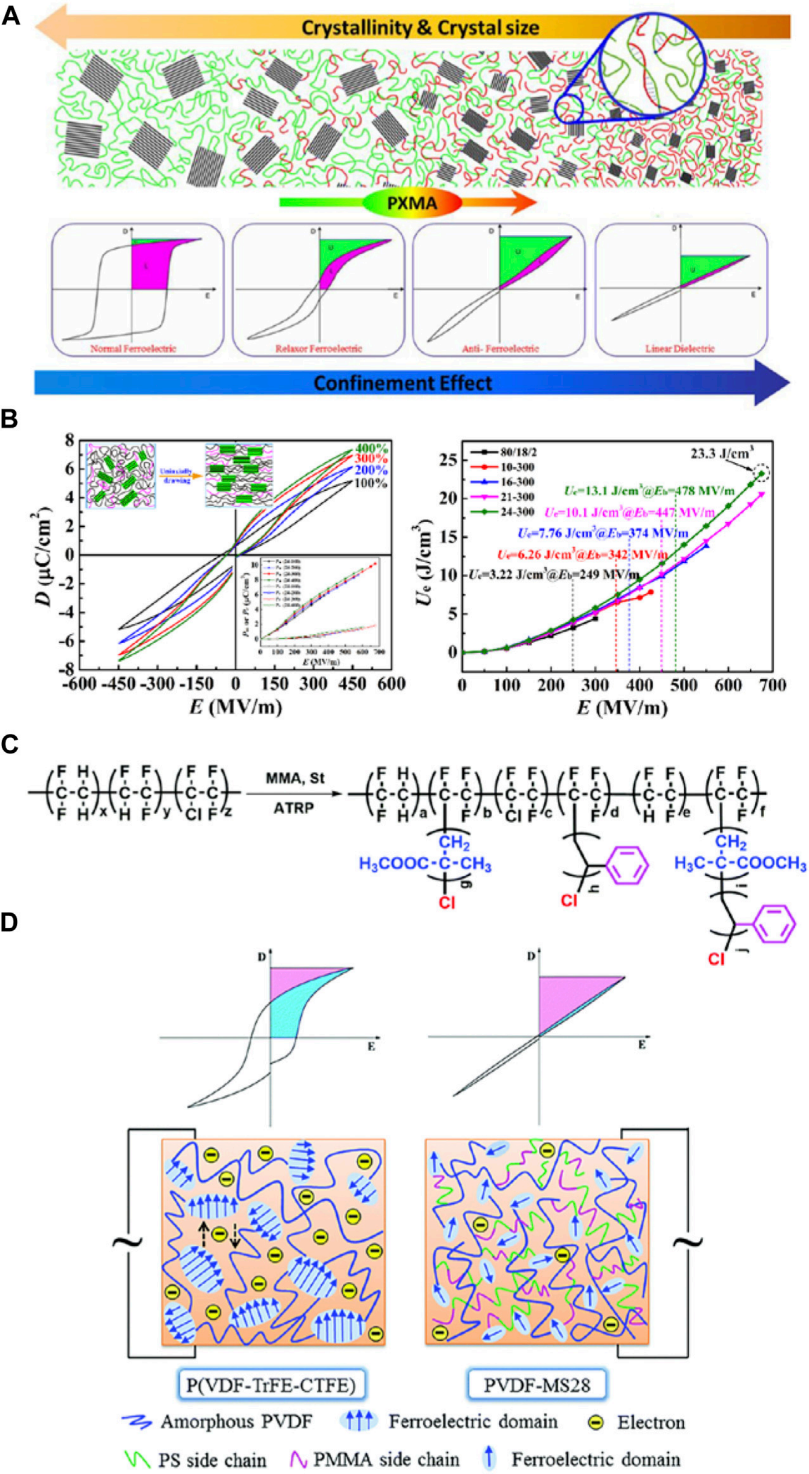
**(A)** Tuning phase transition and ferroelectric properties of P(VDF-TrFE) *via* grafting with desired poly (methacrylic ester)s as side chains; **(B)** High-field antiferroelectric-like behavior in uniaxially stretched P(VDF-TrFE-CTFE)-*g*-PMMA films with high energy density; **(C)** Synthesis of PVDF-MS copolymers using ATRP; **(D)** Schematic models of the molecular morphology of P(VDF-TrFE-CTFE) and PVDF-MS28. Reproduced with permission from Ref. 68, 70, and 71. Copyright 2013, 2016, and 2019 Royal Society of Chemistry.

Zhicheng Zhang’s group has also done a lot of work on high energy storage dielectrics based on PVDF-based modified fluoropolymer using ATRP. In 2012, they reported a novel antiferroelectric-like performance dielectrics P(VDF-TrFE-CTFE)-*g*-PEMA at high poling fields. The grafting copolymers were synthesized following typical ARGET-ATRP procedure using Cu as reductive agent. The *U*_e_ (14 J/cm^3^@550 MV/m) of the resultant grafting polymer effectively improved and that of energy loss dramatically reduced (Li et al., 2012). Then three sets of poly (methacrylic ester)s including PMMA, PEMA and PBMA grafted P(VDF-TrFE-CTFE) copolymers were synthesized *via* ARGET-ATRP and carefully characterized. The *D*-*E* hysteresis behaviors of the graft copolymers could be tuned from typical ferroelectric to either antiferroelectric or linear shape under high electric field (Figure 4A). Meanwhile, significantly reduced energy loss and effectively improved *η* were obtained (Li et al., 2013; Li et al., 2014). Furthermore, a series of P(VDF-TrFE-CTFE)-*g*-PMMA with different PMMA content were synthesized *via* ATRP. All the grafted copolymer films were prepared by a solution-casting process followed by uniaxially stretching with varied extension ratios. Both the content and size of the crystalline and ferroelectric phases improved after uniaxially drawing. Thanks to the strong confinement of rigid PMMA and the alignment induced high breakdown strength, the antiferroelectric-like behavior could be retained up to 675 MV/m with high *U*_e_ (23.3 J/cm^3^) (Figure 4B) (Gong et al., 2016). After that, the same group successfully synthesized a series of antiferroelectric-like P(VDF-TrFE-CTFE)-*g*-MS copolymers using ATRP procedure (Figure 4C). The merits including the excellent miscibility of PMMA with both PSt and PVDF, and the promising insulating performance of PSt segments are combined in the resultant copolymers. The introduction of increased MS side chains leads to significantly enhanced *U*_e_, low conduction loss and energy loss compared with the pristine P(VDF-TrFE-CTFE) (Figure 4D). The highest *U*_e_ of 17 J/cm^3^ and the *η* of 87% at 600 MV/m are achieved by the sample containing 28 wt% MS (Liu et al., 2019b).

**FIGURE 5:**
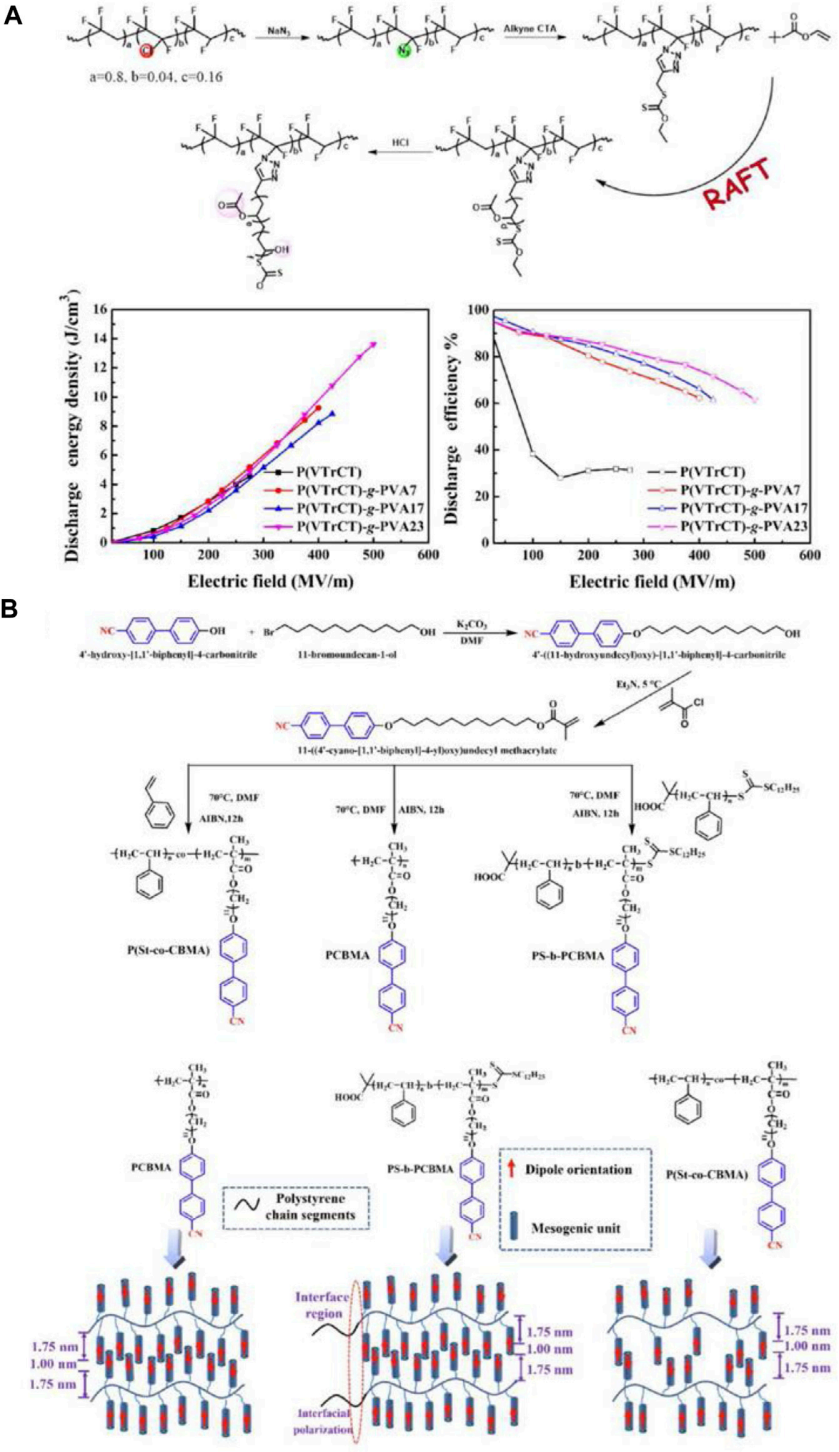
**(A)** Synthesis of P(VTrCT)-*g*-PVA by RAFT polymerization, and *U*_e_ and *η* of P(VTrCT)-*g*-PVA and pristine P(VTrCT); **(B)** The preparation routes of monomers and polymers, and dielectric mechanism of PCBMA, PS-*b*-PCBMA and P(St-*co*-CBMA). Reproduced with permission from Ref. 72 and 73. Copyright 2021 and 2022 American Chemical Society and Elsevier.

In addition, Zhicheng Zhang’s group fabricated a series of P(VDF-TrFE-CTFE)-*g*-PVA with high *U*_e_ and low dielectric loss for using RAFT procedure (Zhang et al., 2021). The PVA side chain shows great compatibility with the PVDF main chain and the hydrogen bond could be constructed among the hydroxyl and ester groups, which is responsible for the suppressed ferroelectric loss and enhanced *E*_b_ and thus improved *U*_e_ and *η*. The graft copolymer containing 23 mol% PVA shows the maximum *U*_e_ of 13.6 J/cm^3^ at 500 MV/m (Figure 5A). This work demonstrates that the hydrogen bond constructed based on the hydroxyl group may offer a strategy to tune the ferroelectric and energy storage performance of PVDF-based fluoropolymers. Hang Luo’s group designed and synthesized several novel PS copolymers with respect to different sequential structure in order to further develop high *ε*_r_ and *U*_e_ of PS dielectrics. Among them, random copolymers (P(St-*co*-CBMA)) were synthesized by free radical polymerization and block copolymers (PS-*b*-PCBMA) were synthesized by RAFT polymerization, respectively (Figure 5B). Due to a large dipole moment (3.9 D) of cyanide group (CN), the high *ε*_r_ value of 4.6 is obtained for PCBMA at 1 kHz and room temperature. Moreover, the dielectric properties of PS-based copolymers can be effectively improved *via* introducing CBMA unit because of the strong orientation polarization, deep traps and good film-forming property (He et al., 2022).

In general, for PVDF-based ferroelectric polymers with high *ε*_r_, although introducing defects into polymers by physical/chemical means can effectively reduce the size of the ferroelectric phase and transform it from a typical ferroelectric to relaxor ferroelectric, antiferroelectric and even linear dielectric, the energy loss can be reduced to less than 20%, but it is still far from the loss of less than 5% for BOPP at a high electric field of 700 MV/m. The high loss of PVDF-based ferroelectric polymers is due to the relaxation of the ferroelectric phase and polar groups, which is determined by its molecular structure and cannot be eradicated. What’s more, PVDF-based fluoropolymers are easily degraded by eliminating HF under high electric fields, which seriously corrodes metal electrodes, which also hinders their application in high-voltage pulse capacitors.

### Dipole glassy polymer for energy storage

In recent years, researchers have paid increasing attention to amorphous glassy polymer dielectrics. There is no difference between the crystalline phase and the amorphous phase of glassy polymers, so the introduction of polar groups will not lead to changes in crystalline properties. More importantly, many glassy polymers have a high *T*_g_, and high expectations are placed on high temperature dielectrics.

Qiming Zhang’s group prepared one polyurea containing aromatic rings in the main chain. The introduction of different contents of polar amino groups made the *ε*_r_ of the polymer adjustable between 4 and 6, and the average *E*_b_ reached 650 MV/m, and *U*_e_ reaches 12.0 J/cm^3^ under this electric field, which is nearly 4 times that of BOPP. In addition, *U*_l_ is limited to about 10% by the strong interaction between N-H bond and C=S bond on the main chain (Wu et al., 2013). Lei Zhu’s group prepared sulfonylated poly (2,6-dimethyl-1,4-phenylene ether) (SO_2_-PPO) by post-functionalization method. The highly polar methylsulfonyl side groups can rotate effectively below *T*_g_ (220°C), and the *ε*_r_ of the polymer is greatly improved through the interaction between polar dipoles, and the *U*_e_ is as high as 22.0 J/cm^3^ under an electric field of 800 MV/m, while the increased *T*_g_ can control the *U*_l_ at a lower level (Zhu et al., 2018). In addition, they synthesized a set of 12 new polyimides (PIs) with one or three polar CN dipoles directly attached to the aromatic diamine part and studied their electric energy storage properties. It was found that adding highly polar nitrile groups to the PI structure increased *ε*_r_ and thus *U*_e_, especially at high temperatures (Treufeld et al., 2014).

Although glass dipole polymer dielectrics are expected to be used under high temperature conditions, the film-forming properties of such polymers containing aromatic rings in the main chain are poor, and some can only form films on substrates with electrodes and perform subsequent tests. It has caused great difficulties in the preparation and subsequent application of large-area flexible films. In addition, for this type of polymer, the dielectric constant is increased by the interaction between dipoles, and the high dielectric loss caused by the relaxation of the dipole polarization is also unavoidable at the same time.

### The application of ROMP in preparation of polymer dielectric

**FIGURE 6:**
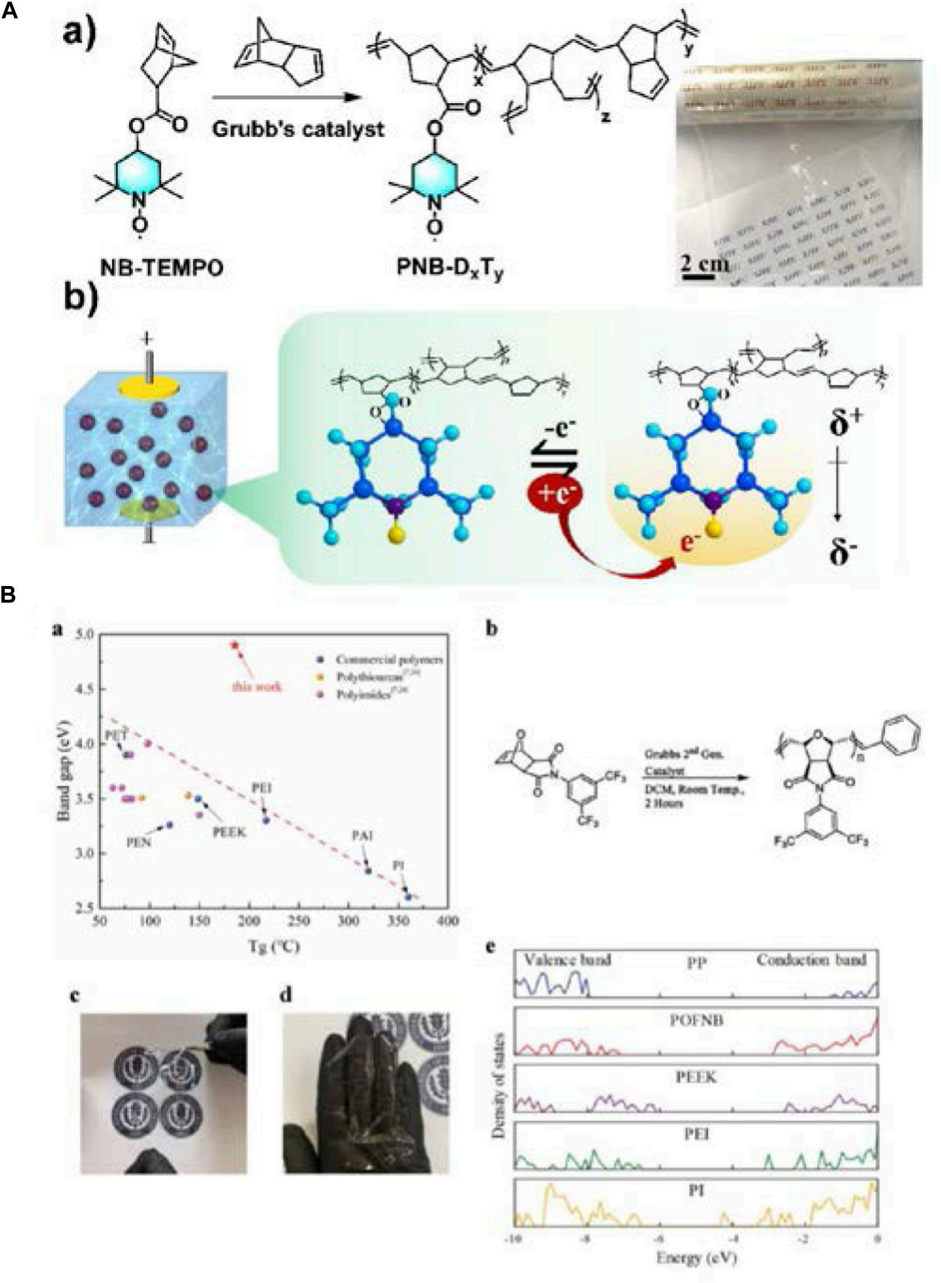
**(A)** Chemical synthetic route and dipole formation mechanism: (a) Chemical synthetic route of PNB-DxTy and dielectric film obtained by one-step polymerization, cross-linking, and solution casting; (b) Single radical capturing electrons under an electric field and the resulting dipole; **(B)** (a) The bandgap versus. *T*_g_ for POFNB and high-temperature polymers with aromatic backbone structure; (b) Schematic of the synthesis process for POFNB; (c,d) Photographs of the free-standing POFNB films; (e) The electronic density of states of POFNB, PP, PEEK, PEI, and PI, computed based on density functional theory. Reproduced with permission from Ref. 74 and 75. Copyright 2020 American Chemical Society and Wiley Periodicals, Inc.

ROMP is also a commonly used method for synthesizing polymer dielectrics. Recently, a novel radical-containing polymeric dielectric material, PNB-D_x_T_y_, with adjustable dipole dispersion was synthesized through a typical one-pot ROMP procedure by Yanfeng Zhang’s group (Figure 6A). The prepared material exhibited high *U*_e_ of 10.6 J/cm^3^ together with promising *η* of 92% at 500 MV/m, and good stability. The PNB-D_x_T_y_ polymer also demonstrated a high *E*_b_ of approximately 700 MV/m. The effective strategy revealed that the isolated stable radical in the low-polarity polymer matrix suppressed the energy loss and created a new paradigm for high-energy and low-loss flexible capacitors (Ma et al., 2020). Yang Cao’s group synthesized an all organic polymer, POFNB, with saturated fused bicyclic structure in the backbone by a ROMP procedure using Grubbs generation two catalyst (Figure 6B). POFNB has a key design feature in that it is a polyolefin consisting of repeating units of fairly rigid fused bicyclic structures and alkenes separated by freely rotating single bonds, endowing it with a large bandgap of ≈5 eV and flexibility, while being temperature-invariantly stable over −160 to 160°C. At 150°C, POFNB features an unprecedented *U*_e_ of 5.7 J/cm^−3^ far outperforming the best reported flexible dielectrics (Wu et al., 2020).

**FIGURE 7:**
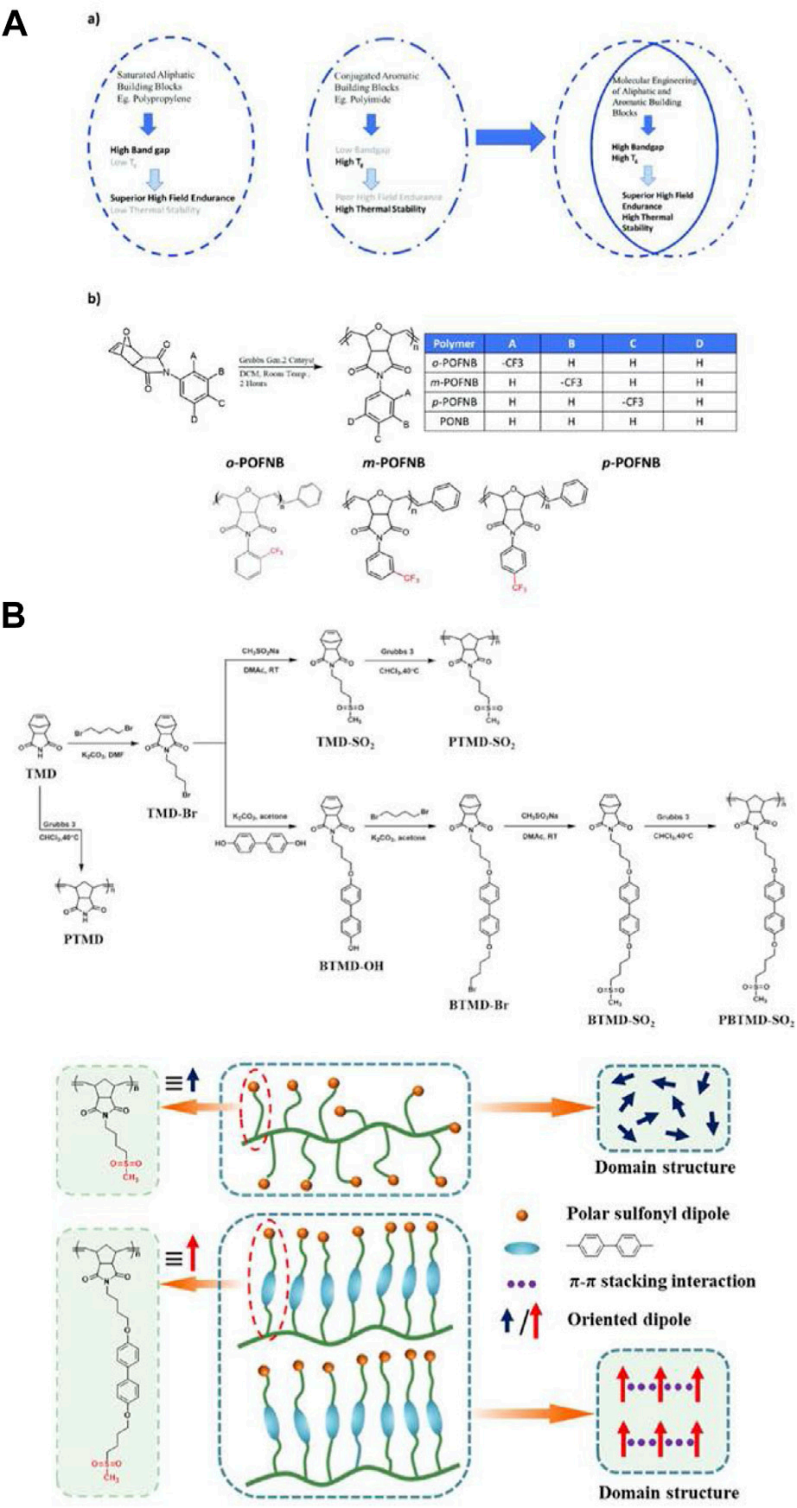
**(A)** Design of high temperature polymer dielectrics: (a) Conceptual schematic for design of high temperature dielectric polymers; (b) Molecular structures of polymers investigated in this work; **(B)** Procedure for the synthesis of monomers and polymers, and schematic illustration of the effect of biphenyl groups on the dielectric properties of polymers. Reproduced with permission from Ref. 76 and 77. Copyright 2022 and 2011 The Royal Society of Chemistry and American Chemical Society.

Deshmukh et al. (2022) prepared a class of POFNB by the versatile ROMP, which is designed by changing the -CF_3_ substitution position on the benzene ring to optimize the electrical and thermal properties (Figure 7A). Wide bandgap, freely rotatable structural groups along with a combination of flexible and rigid moieties, achieved through a molecular engineering approach and verified using computational methods resulted in a class of polymer dielectrics with the highest ever discharged energy density (6.5 J/cc) up to 200°C. The uncovered polymer design strategy introduces a platform for high performance dielectric development for extreme thermal and electric field conditions. In order to improve the *ε*_r_ and *U*_e_ of polymer dielectrics, Xu et al. (2021) synthesized sulfonyl-containing poly (norbornene)s with and without biphenyl groups (denoted as PBTMD-SO_2_ and PTMD-SO_2_) by ROMP procedure (Figure 7B). The *ε*_r_ of PBTMD-SO_2_ with the biphenyl side groups was as high as 11.1 at 25°C and 1 kHz, which was nearly 35% higher than that of PTMD-SO_2_ due to the stronger orientational polarization through the incorporation of the π−π stacked biphenyl side groups in polymers. This work provides a new idea for the molecular design of polymer dielectrics with high *ε*_r_ and low tanδ.

**FIGURE 8:**
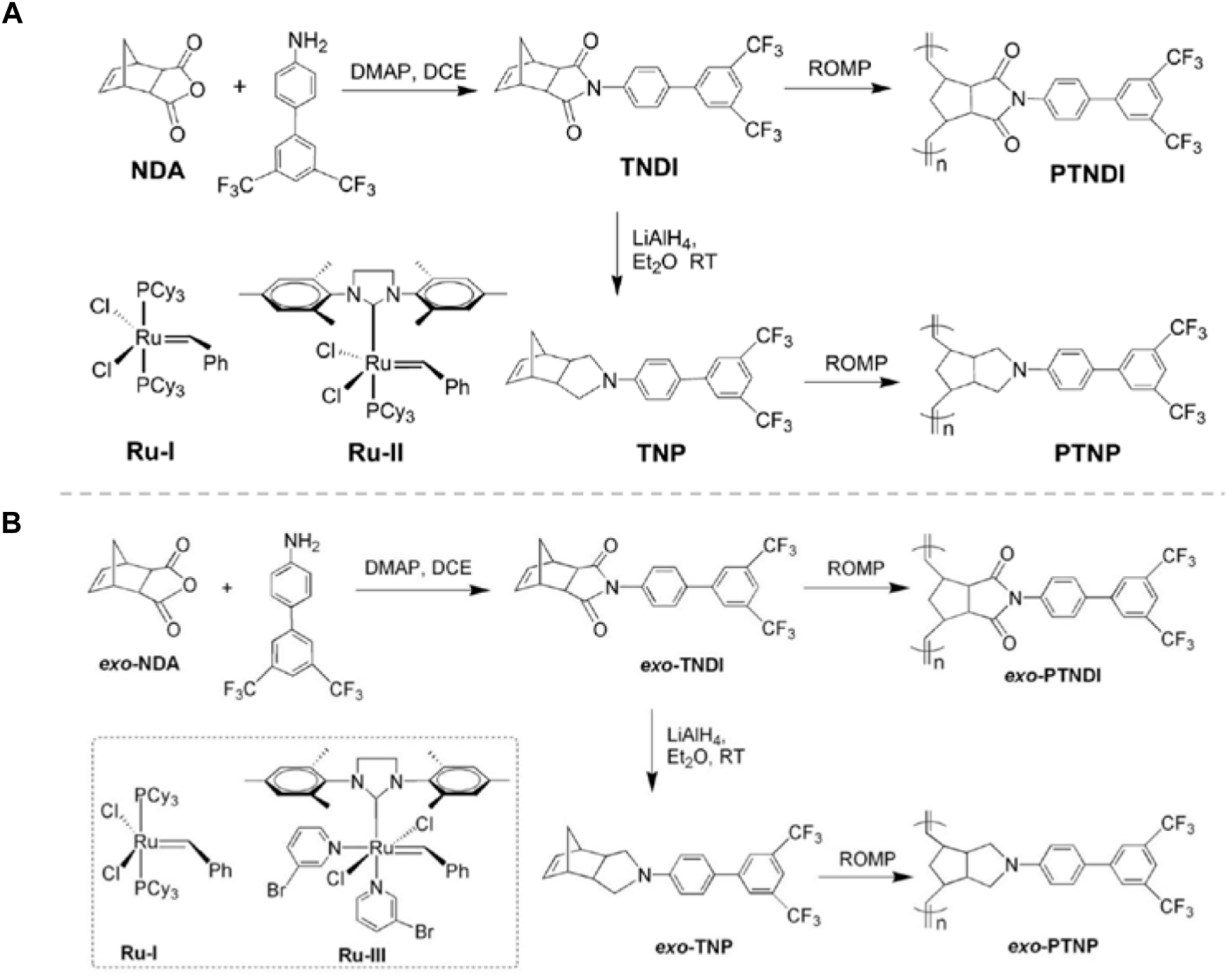
Synthesis of functional monomers **(A)**, exo-monomers **(B)** and polymers. Reproduced with permission from Ref. 78 and 79. Copyright 2013 Wiley Periodicals, Inc.

In recent years, Meiran Xie’s group has done a series of researches about the application of ROMP in the synthesis of polymer dielectrics. In 2013, they successively synthesized functional PNBEs, and *exo*-PNBEs containing pyrrolidine moiety and bis(trifluoromethyl) biphenyl side groups, named as PTNP, PTNDI, *exo*-PTNP and *exo*-PTNDI, by ROMP using different Grubbs’ catalysts (Figure 8) (You et al., 2013a; You et al., 2013b). Polymers with different chain microstructure have different dielectric properties. The authors believe that the pyrrolidine moiety is an essential element to control the stereoregularity of polymers. And The results demonstrated that both the steric effect of catalysts and the configuration of monomers have a significant impact on the microstructure and properties of polymers.

**FIGURE 9:**
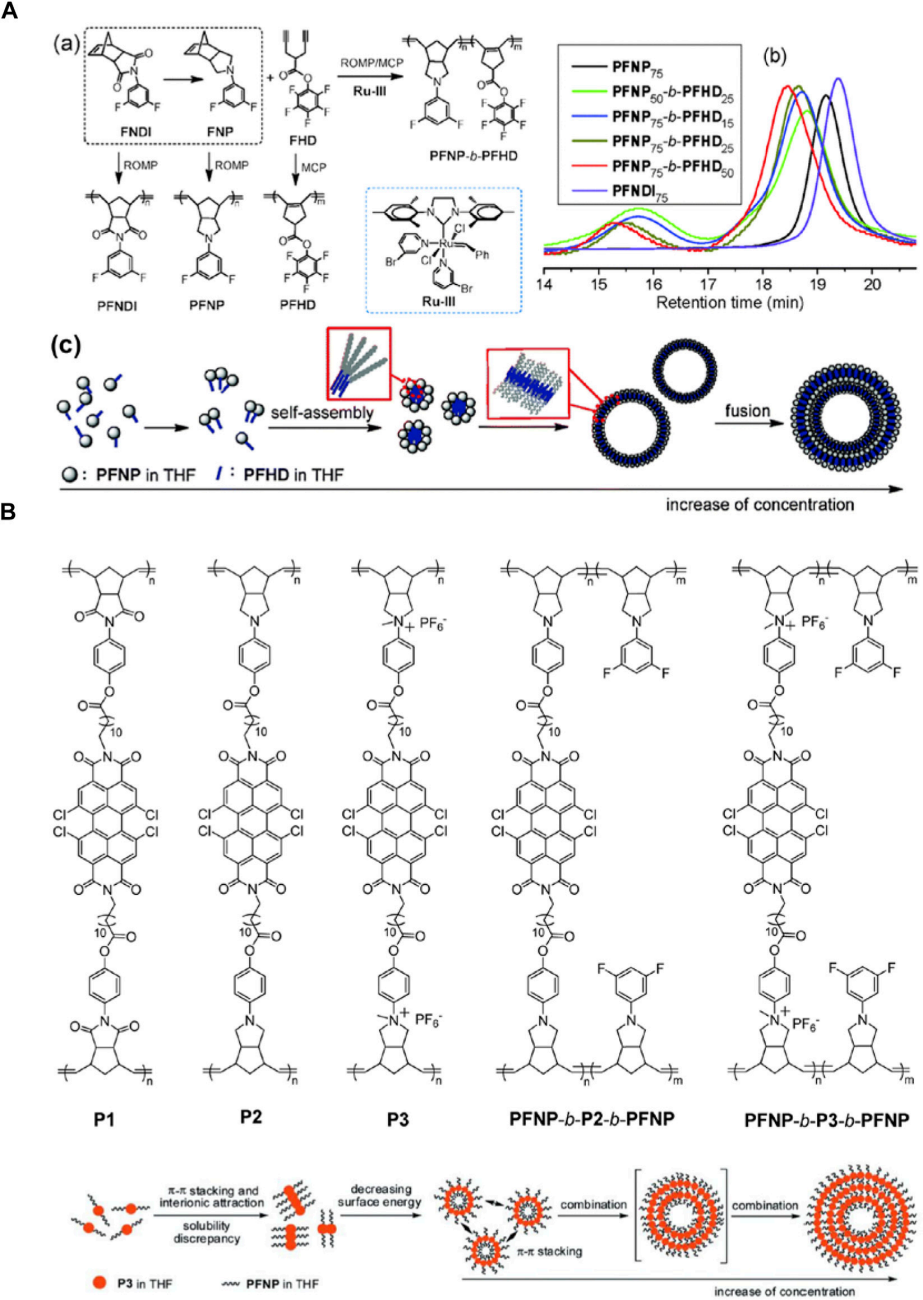
**(A)** Synthetic routes (a) and GPC traces (b) of the homo- and copolymers; (c) the schematic process of hollow sphere nanostructure formation from a single chain of the copolymer in THF; **(B)** Structure of polymers obtained by ROMP method and the schematic process of a tree ring-like nanostructure by the self-assembly of triblock copolymer in THF. Reproduced with permission from Ref. 80 and 81. Copyright 2015 and 2016 The Royal Society of Chemistry.

In 2015, they synthesized polymers of poly (*N*-3,5-difluorophenyl-norbornene-dicarboximide) (PFNDI), PFNP, PFHD, and PFNP-*b*-PFHD by ROMP, MCP, and tandem ROMP-MCP procedure, respectively. And they reported for the first time a dielectric-percolative block copolymer of PFNP-*b*-PFHD, which could self-assemble into unique nanostructures of micelles or hollow spheres (Figure 9A). The block copolymer exhibits a high *ε*_r_, low dielectric loss, and high *U*_e_ due to the strong dipolar and nano-interfacial polarization contributions (Liu et al., 2015). Subsequently, they prepared functional poly (bisnorbornene)-based ladderphanes, P1, P2, and P3, as well as the derived triblock copolymers containing two blocks of poly (*N*-3,5-difluorophenyl-norbornene pyrrolidine) (PFNP), PFNP-*b*-P2-*b*-PFNP and PFNP-*b*-P3-*b*-PFNP by ROMP (Figure 9B). The ionic copolymer PFNP-*b*-P3-*b*-PFNP could self-assemble into a unique tree ring-like nanostructure, in which the ionic P3 blocks were isolated between the insulating PFNP blocks, and the long distance migration of ions was difficult to achieve, resulting in lower dielectric dissipation and higher *η* than those of the ionic homopolymer P3. This research presented a practical way to improve the dielectric properties by combining the ionic, dipolar, and nano-interfacial polarizations as well as the stereoregular chain microstructure (Chen et al., 2016). They synthesized another ionic poly (bisnorbornenepyrrolidium) homopolymer ladderphanes and the derived triblock copolymers containing two insulating polar blocks by ROMP. The copolymers had a lower dielectric dissipation and higher *η* than those of ionic homopolymer because of the insulating blocks between ionic blocks (Chen and Xie, 2017).

In the following years, the same research group continued to use the ROMP method to prepare a series of block copolymers with similar structures, and studied their morphologies and dielectric energy storage properties after self-assembled. In 2017, a block copolymer consisting of functional PNBE and polyacetylene (PA) segments was synthesized and self-assembled into a superhelical nanotube morphology from achiral building blocks (Liu F. et al., 2017). Then, the double-stranded block copolymers containing ionic conductive and strong polar segments were synthesized and self-assembled into the hollow sphere nanostructure (Chen et al., 2017). In 2018, novel high-*k* and low loss bis(double-stranded) block copolymers, containing the ionic-conjugated hybrid conductive segments (HCS) with narrow band gap and the insulating segments with wide band gap were synthesized (Chen et al., 2018a). And in 2019, disubstituted pendant-functionalized block copolymer consisting of insulating PNBE and conductive PA segments was synthesized. These copolymers all displayed high *ε*_r_, low dielectric loss, enhanced *U*_e_ and good *η* (Zhou et al., 2019). At the same year, block copolymers with push-pull azobenzene pendants were synthesized and could self-assemble into core-shell nanostructures with high dipolar and interfacial polarizations, which were contributed by the strong polarity of azobenzene pendants bearing both electron-donating pyrrolidine and electron-withdrawing trifluoromethyl or nitro groups, and by the unique nanostructure of polymers, respectively (Zhu et al., 2019). The excellent dielectric and energy storage capability were attributed to the unique macromolecular structure and well-defined nanomorphology, which not only enhanced the dipolar, electronic, and interfacial polarizations but also significantly suppressed the leakage current and increased the *E*_b_ by wrapping the narrow band gap segments in the wide band gap segments. These strategies provided a new way for the development of advanced nanodielectric materials. In addition, they first synthesized block copolymer using ROMP, and then modified copolymer by 1,2,4-triazoline-3,5-dione (TAD) in a controlled manner by tuning the TAD feeding amount (Chen et al., 2018b).

In general, the all-organic polymer dielectric prepared based on ROMP has a rigid main chain skeleton and can introduce polar groups through side groups. This kind of material has diverse structures, moderate *U*_e_, low loss, and the cross-linked film has improved *E*_b_ and has a certain toughness. Further research can be done to prepare dielectrics with novel structures through monomer design.

## Construction of all-organic polymeric dielectric composites by structural design

Recently, topologically structured composites were rationally designed to combine synergistically the complementary properties of the spatially organized constituent layers. Topologically structured composites include an insulating layer to suppress conduction loss and avoid early breakdown under high electric fields due to the blocking effects of the interfaces between the neighboring layers, and have been widely used in the field of electrical insulation (Shen et al., 2015; Jiang et al., 2016). It has been demonstrated experimentally that insulation layers with high *E*_b_ when placed near the electrodes can effectively impede the charge injection and introduce deep traps for charge carriers (Chen et al., 2018c). In dielectric materials, there are means to improve the properties of dielectric materials (e.g., dielectric constant, breakdown electric field) through constructing multi-layer structures, hydrogen bonds and cross-linking structures, or by regulating the interface structure and crystal morphology, etc. And we will make a brief summary of the above methods.

### Multilayer-structured all-organic polymer films

Inspired by the multilayer structure in nature (e.g., abalone shells, peacock feathers, and butterfly wings), a variety of artificial multilayer structural materials have been successfully prepared and proven often have superior properties. Therefore, designing multilayer structural materials have become a promising method to improve the performance of dielectrics and has played a very important role in the exploitation of high energy-storage performance dielectrics in particular (Feng Q. K. et al., 2021). In the past 10 years, remarkable progress has been made in the preparation of organic-inorganic composite dielectric materials by doping ferroelectric ceramics into polymers because the *ε*_r_ is positively correlated with the *U*_e_ (Shen et al., 2015; Jiang et al., 2016; Liu W. et al., 2017; Chen et al., 2018d; Wang et al., 2018) However, this method often leads to an increase in dielectric loss and a decrease in energy storage efficiency due to the poor dielectric matching and compatibility between the two phases. In addition, due to the brittleness and high density of ceramic materials, it brings a lot of inconvenience to the processing and miniaturization of capacitors. Therefore, the preparation of all-organic polymer dielectric films with layered structure through topology design has attracted the attention of domestic and foreign researchers.

**FIGURE 10:**
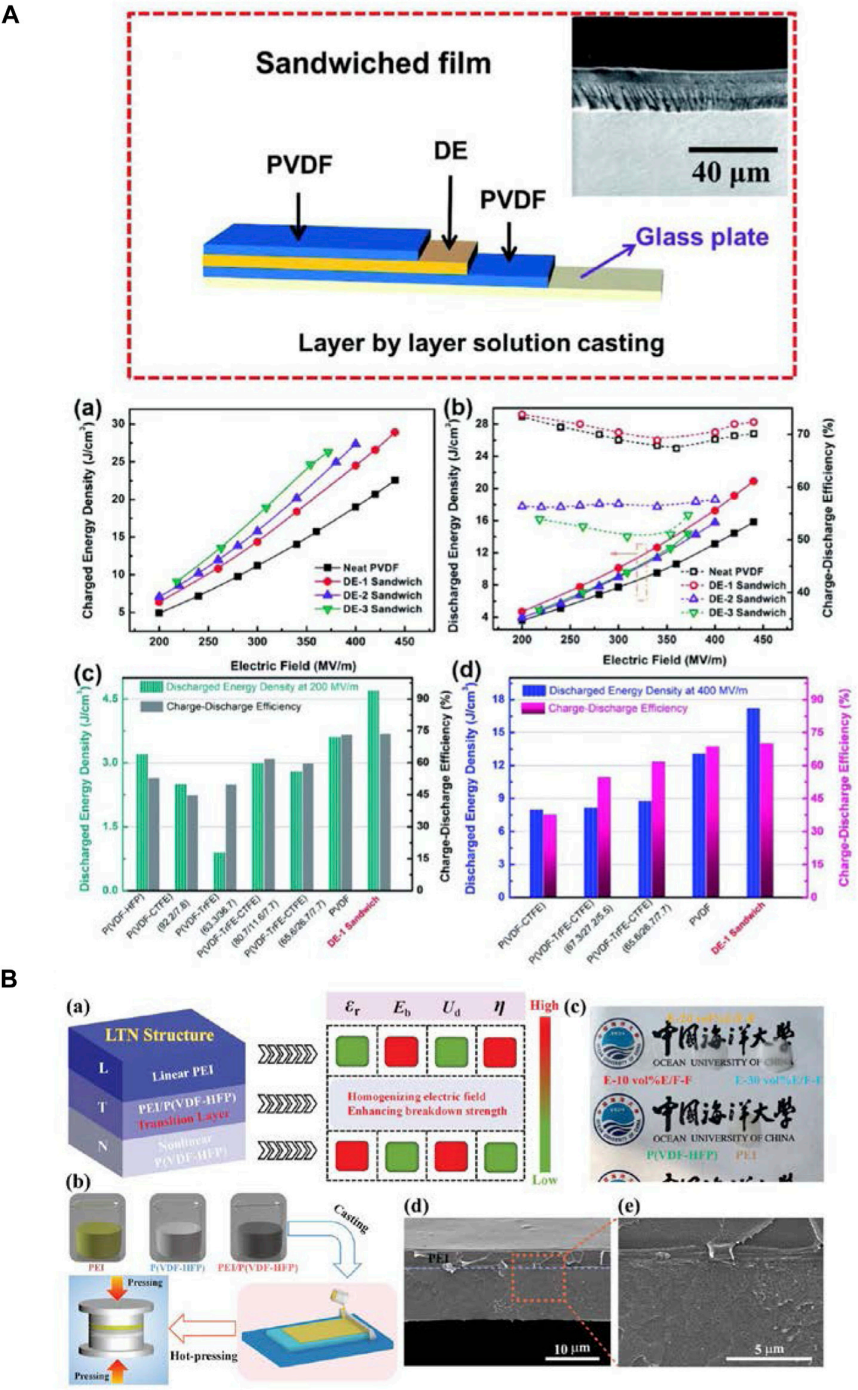
**(A)** Schematic illustration of the sandwich-structured films through the layer-by-layer solution casting method, and (a) Charged energy density, (b) Discharged energy density and charge-discharge efficiency of pristine PVDF and the sandwich-structured polymer films as a function of the applied field, Comparison of the discharged density and charge-discharge efficiency of the DE-1 sandwich polymer film at (c) 200 MV/m and (d) 400 MV/m with PVDF and its copolymer and terpolymer films. **(B)** (a) Schematic illustration of the dielectric energy-storage characteristics of the asymmetric LTN structure; (b) Fabrication process of the single-layer and trilayer composites; (c) Photograph of pure PEI, pure P(VDF-HFP), and trilayer composites; (d,e) Cross-sectional SEM morphology of E-10 vol%E/F-F trilayer composite. Reproduced with permission from Ref. 91 and 97. Copyright 2018 and 2021 The Royal Society of Chemistry, and Wiley.

**FIGURE 11:**
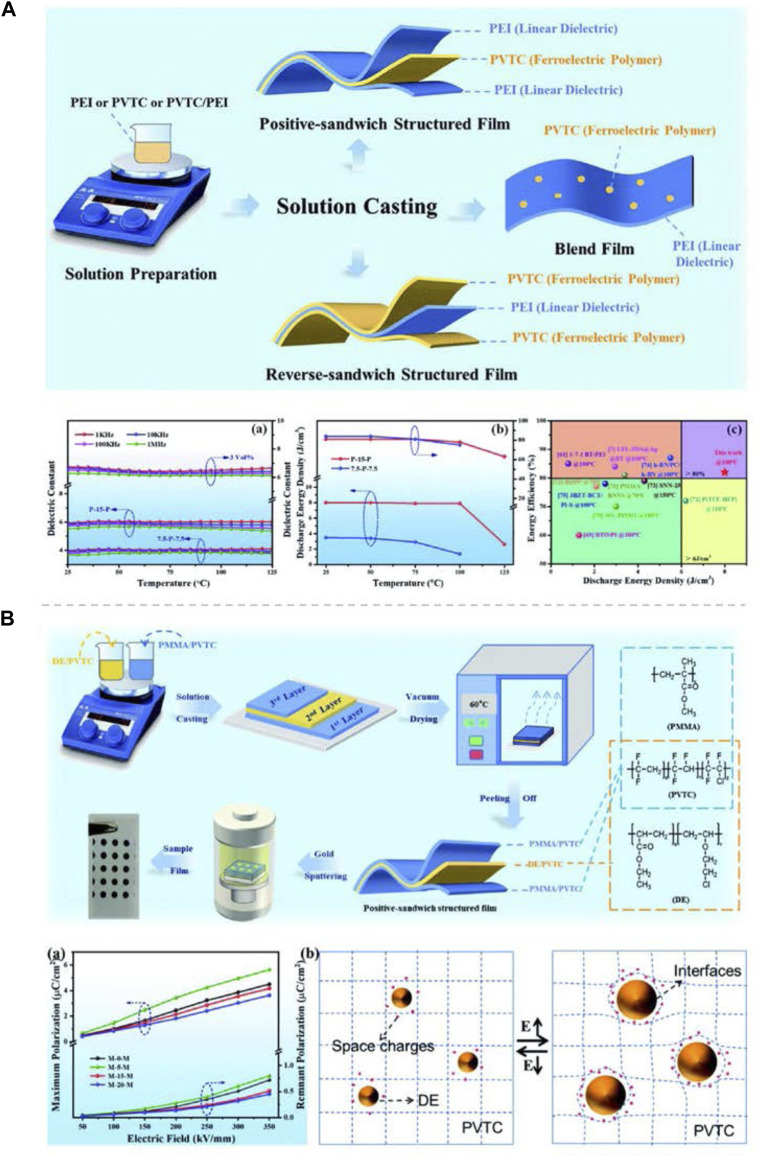
**(A)** Schematic of the positive-sandwich structured, reverse-sandwich structured and polymer blend films fabrication process, and (a) temperature dependence of the dielectric properties of the different structures film at different frequencies; (b) temperature dependence of the *U*_e_ and *η*. **(B)** Flow chart showing the preparation of the multilayer dielectric polymer films, and (a) *D*_max_ and *D*_r_ of the positive-sandwich-structured films as a function of the electric field; (b) Polarization mechanism of DE in the PVTC matrix. Reproduced with permission from Ref. 99 and 100 Copyright 2021 and 2022 The Royal Society of Chemistry.

Hong Wang’s group has done lots of work on sandwich and multilayer structure of all-organic polymer dielectric composites. In 2018, they reported the design and preparation of all-polymer sandwich structured films comprised of two outer layers of PVDF to provide high breakdown strength and an interlayer of acrylic rubber dielectric elastomers (DEs) to offer high electric displacement (Figure 10A). The energy storage performance of the sandwich architecture films can be significantly improved by modulating the thickness of DEs, as confirmed by the *D*-*E* loops and leakage current measurements. Markedly enhanced electric displacement and *E*_b_ have been achieved in the sandwich structured films with an optimum DE central layer thickness of 4 μm, which leads to an ultrahigh *U*_e_ of 20.92 J/cm^3^ and a high *η* of 72%, by far the highest values ever achieved in all-polymer dielectrics. The spatial organization of the DE into the sandwich structures provides an effective way for achieving the high energy storage capability for flexible energy storage devices (Chen et al., 2018a). Subsequently, they prepared multilayered polymer dielectric films based on the incorporation of low-*ε*_r_ PMMA and high-*ε*_r_ ferroelectric polymer *via* the establishment of multiple interlaminar interfaces and modulation of component ratios. The trilayered all-polymer film with optimized component ratio is capable of operating with 84% *η* and concurrently delivering 20.3 J/cm^3^ energy density along with excellent mechanical properties. This work suggests great potential of the multicomponent ferroelectric polymers with layered architecture for electrical energy storage applications (Chen et al., 2018b). Later, the group published a mini review about the research advances of multilayered hierarchical polymer composites (MHPCs), including inorganic particle/organic MHPCs and all-organic layered films in the field of high-energy-density capacitors. The efficient strategies for improving the energy storage performance of MHPCs have been highlighted (Wang et al., 2019). In 2019, they fabricated another series of all-polymer sandwich-structured films comprised of PMMA dispersed in ferroelectric P(VDF-HFP) as the outer layer and PMMA as the middle layer. And a high *U*_e_ of 11.8 J/cm^3^ along with a very high efficiency of 89% has been achieved in the sandwiched film with 30 wt% DE at an applied field of 300 MV/m. All these features offer new opportunities for designing a new class of hierarchically structured dielectric polymers with excellent energy storage capability that can be achieved at relatively low electric fields (Chen et al., 2019). Then, targeting at achieving simultaneous high *U*_e_ and high *η*, the unique design of asymmetric all-polymer trilayer composite consisting of a transition layer sandwiched by a linear dielectric layer and a nonlinear dielectric layer was reported by the same group (Figure 10B). It is demonstrated that the nonlinear dielectric layer offers high *U*_e_, while the linear dielectric layer provides high discharge efficiency. Especially, the transition layer can effectively homogenize the electric field distribution, resulting in greatly elevated *E*_b_ and improved *U*_e_ (Sun L. et al., 2021). Figure 11.

Dou Zhang’s group has also done some work on the all-organic multilayer-structured dielectric polymer films consisting of linear dielectric and ferroelectric polymers made by simple layer-by-layer solution casting. In 2020, they prepared multilayer-structured composites, PVDF and P(VDF-TrFE-CTFE) (PVCT) used as different layers, with excellent dielectric and energy storage properties by the stacking method and studied the effect of layer numbers on the performance of the composites. The multilayer structure and the formed interfaces could suppress carriers’ movement; therefore, the composites maintained a low dielectric loss and leakage current density (Xie et al., 2020). In 2021, they fabricated series of positive-sandwich structured films, with the central PVTC layer and the outer PEI layers, reverse-sandwich structured films and single-layered blend films for optimization and comparison (Figure 11A). The influence of the linear/ferroelectric volume ratio and the polymer film structure on the energy storage performance has been thoroughly researched. Experimental results showed that the all-organic positive-sandwich structured film exhibits a maximum *U*_e_ of 8.0 J/cm^3^, which was more than two times that of the pure PEI film. And more importantly, it possesses excellent temperature stability of the dielectric and energy storage properties from 25^o^C to 100°C (Wang et al., 2021). Then, they used the same method to prepare a series of films by selecting ferroelectric PVTC as the polymer matrix and polyacrylate elastomer (DE) and PMMA as organic fillers (Figure 11B). The redistributed electric field in the sandwich-structured films could effectively prevent the development of an electric tree and avoid early breakdown of the dielectric, coupled with adopting the more insulated 20 wt%-PMMA/PVTC layer as the outer layers to prevent charge injection and electrical conduction, which led to a greatly enhanced *E*_b_, limited leakage current density and improved *U*_e_ in the positive sandwich-structured film with the optimized DE content. These works provide a potential pathway to develop advanced polymer capacitors for various electric applications based on ferroelectric polymers with a layered architecture and organic elastomer fillers (Wang C. et al., 2022).

Sun S. et al. (2021) designed a novel all-polymer trilayer structure, where the PMMA was used as the top layer to obtain a high *η*, and ferroelectric P(VDF-HFP) was employed as the bottom layer to obtain a high *U*_e_. Particularly, the PMMA/P(VDF-HFP) blend composite is used as the middle layer to homogenize the electric field inside the trilayer composites, turning out an obviously boosted *E*_b_ and elevated *U*_e_. The result shows that the design strategy furnishes us with a groundbreaking pathway to obtain dielectric polymer capacitors with simultaneous high *η* and *U*_e_. Guang Liu et al. prepared a symmetrical sandwich structure by compounding PC and PVDF, in which PC has higher insulation and heat resistance than PVDF. It was found that as the temperature increases, the applied electric field would gradually concentrate on the PC layer, while the electric field borne by the PVDF layer gets much lower. In this way, a similarity-intelligent dielectric with the performance of self-adjusting electric field distribution was obtained, which can effectively avoid premature breakdown of PVDF under high-temperature conditions. This work effectively improves the high-temperature energy storage characteristics of PVDF and broadens its application fields (Liu et al., 2021).

Generally, the layered all-polymer films always possess higher *E*_b_ compared with the single-layered counterparts. Through a rational design of the layered structures, the *U*_e_ can be significantly enhanced by ∼100% in the all-polymer films compared with traditional single-layered films. However, not all polymers could be molded by dissolution in a solvent, especially for the engineering polymers targeted for high-temperature applications. Moreover, it should be noted that the microscopic characteristics of the layer interface in different multilayer systems are different, the study of the relationship between the interface and the electrical properties needs to be treated differently. Therefore, there is still room for performance improvement and theoretical development for multilayer dielectric.

### Hydrogen bond and cross-linked structures

**FIGURE 12:**
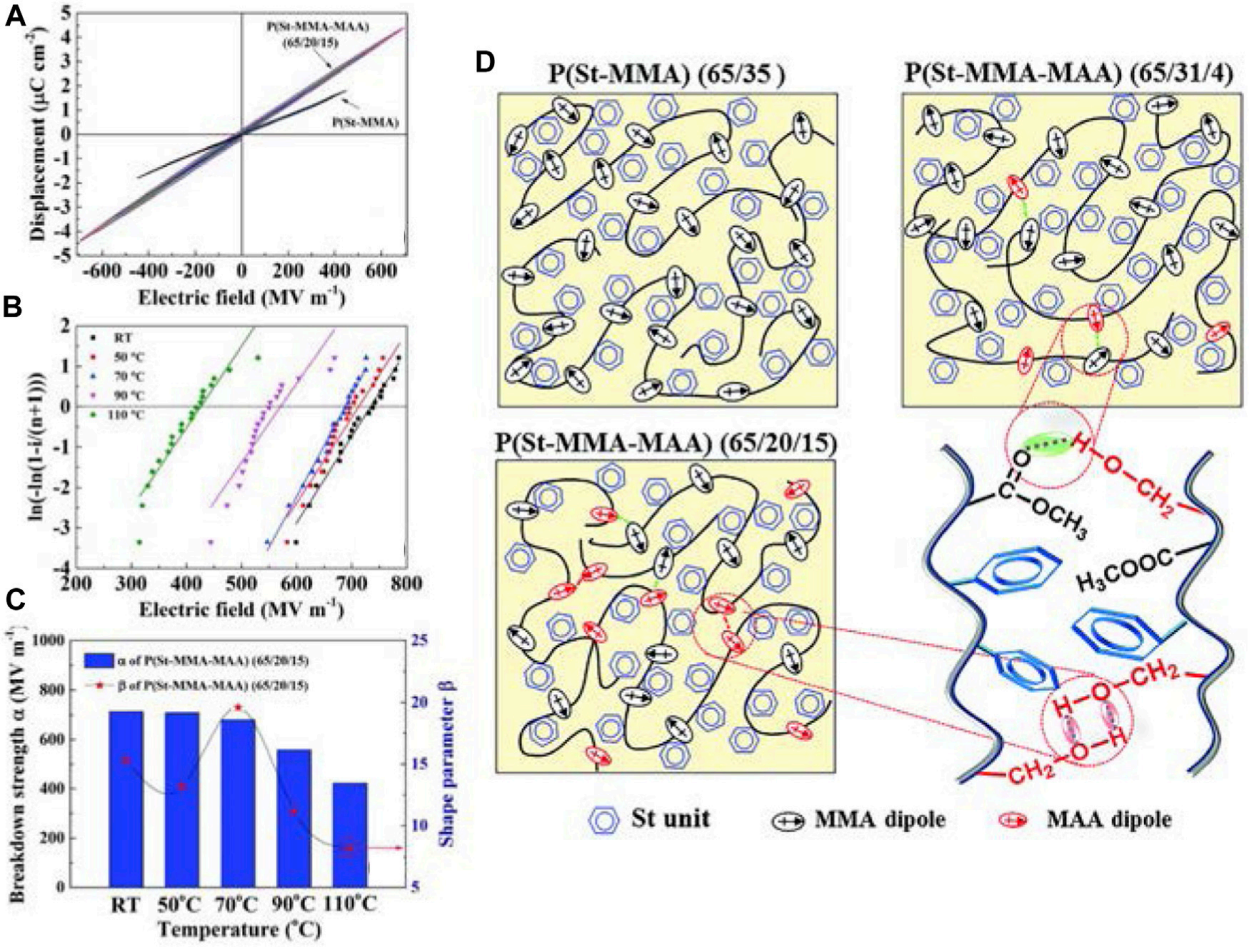
**(A)** *D*-*E* loops of P(St-MMA) and P(St-MMA-MAA) (65/20/15) at room temperature, **(B)** *E*_b_ and **(C)** the Weibull distribution of P(St-MMAMAA) (65/20/15) at elevated temperature, **(D)** Schematic models of the molecular morphology of P(St-MMA) and P(St-MMA-MAA)s. Reproduced with permission from Ref. 104. Copyright 2019 The Royal Society of Chemistry.

In recent years, the construction of hydrogen bonds and cross-linked structures are also commonly used methods to improve the breakdown electric field of polymer dielectrics. Regarding the construction of hydrogen bonds, Zhicheng Zhang’s group has already done some work. In 2016, they firstly reported the synthesis of P (MMA-MAA) copolymers *via* the partial hydrogenation of PMMA. The introduction of -OH groups leads to the formation of H-bonds among -OH and ester groups, which is responsible for the enhanced *T*_g_ and Young’s modulus. As a result, both the *ε*_r_ and *E*_b_ have improved significantly. Therefore, constructing strong H-bonds in glassy dipolar polymers might offer a great option for designing and fabricating polymeric dielectrics with high *U*_e_ and low *U*_l_ (Wang Z. et al., 2016). In 2019, they prepared P(St-MMA-MAA) terpolymers from P(St-MMA) using the same method of partially hydrogenating MMA units. The benzyl groups of the low-polarity polystyrene matrix suppressed the aggregation of polar units under high energy fields, i.e., diminishing the aggregation caused energy loss in the capacitive terpolymers. H-bonds between -OH on MAA units and ester groups on MMA units could favor the enhancement of *T*_g_, *ε*_r_, Young’s modulus, and *E*_b_ of polymers (Figure 12). Besides, the excellent performance of P(St-MMA-MAA) films is preserved at temperatures up to 110°C. This strategy creates a new pathway to prepare glassy dielectric polymer films for application in flexible, efficient energy storage devices used in harsh environments such as high electric fields and elevated temperature (Liu et al., 2019a). In the previous section, it was introduced that the same research group prepared a series of P(VDF-TrFE-CTFE)-*g*-PVA using RAFT procedure. And H-bond could be constructed among the -OH and ester groups, which is responsible for the suppressed ferroelectric loss and enhanced *E*_b_ and thus improved *U*_e_ and *η* (Zhang et al., 2021).

Wang R. et al. (2022) reported a kind of all-organic ultrahigh-energy-density dielectric composites materials consisting of ferroelectric polymer P(VDF- HFP) and glucose, exhibiting record *U*_e_ (37.7 J/cm^3^) that outperforms the state-of-the-art dielectric polymer composites. The glucose molecules rich in -OH groups are found to facilitate the formation of a physically cross-linking network of hydrogen bonds within the polymer matrix, which lead to increased crystallinity, reduced crystallite size, and stabilized *γ* phase, and hence improved electric displacement and mitigated hysteresis loss of the polymer. Both simulation and experimental results show that the hydrogen bonds serve as the trapping sites of charge carriers and suppress conduction loss. Yuan C. et al. (2020) present an innovative approach to substantially improve the thermal stability and concurrently reduce the dielectric loss of PP. In particular, cross-linkable antioxidant groups, hindered phenol (HP), are incorporated into PP *via* well-controlled chemical synthesis. The grafted HP can simultaneously serve as radical scavenger and cross-linker, thereby constraining thermally decomposed radicals and charge transport in the synthesized PP-HP copolymer. PP-HP after aging at 190°C exhibits a better dielectric performance than the PP without any substantial thermal treatment. The promising results imply that the copolymers could address the low thermal stability of PP and replace PP for advanced power systems where high repetition rates and elevated operating temperatures are required.

In addition to improving the *E*_b_ of polymer dielectrics, the crosslinking strategy has also been regarded as one of the most feasible approaches for polymer dielectrics to meet the high temperature requirements. Khanchaitit et al. (2013) prepared cross-linked P(VDF-CTFE) ferroelectric polymer networks by hot-pressing the mixture of P(VDF-CTFE), 1,4-bis(t-butyl peroxy) diisopropylbenzene as the initiator and triallyl isocyanurate (TAIC) as the cross-linking agent. It is believed that the Cl groups in P(VDF-CTFE) are abstracted to form macromolecular radicals that are then cross-linked with TAIC. The results showed that the ferroelectric polymer networks exhibiting significantly reduced dielectric loss, superior polarization and greatly improved *E*_b_ and reliability, while maintaining their fast discharge capability at a rate of microseconds and enabling the operation of the ferroelectric polymers at elevated temperatures. Tang et al. (2021) have summarized recent progress in the field of crosslinked polymer-based materials as high-performance dielectrics at elevated temperature. And self-crosslinking polymers, polymers crosslinked by agents and crosslinked polymer nanocomposites were the focus of materials reviewed. The authors believe that the crosslinking strategy has just opened a window in seeking superior high temperature energy storage applications, and there is a long way to go.

Generally, it significantly could reduce dielectric loss and dielectric relaxation and increase *E*_b_ and mechanical properties through the construction of hydrogen bonds or cross-linked structures. In addition, these strategies shed light on ways for the fabrication of dielectric polymers and polymer nanocomposites working at high temperature. For cross-linked polymers, film processing is an area that requires special consideration later on. In order to apply the film to actual capacitors and realize mass production, it is important to develop advanced film-forming technology to obtain thin films with uniform thickness.

### Interface and crystal morphology regulation

Controlling the aggregated structure of the molecular chain, that is, the crystalline morphology, or regulating the interface between the electrode and the dielectric material is also a commonly used method to improve the energy storage performance of polymer dielectric materials in recent years. Miranda et al. (2017) used a model semicrystalline polymer, poly (ethylene naphthalate) (PEN) to examine the effect of crystalline/amorphous lamellar microstructure on amorphous chain relaxations and how this affects dielectric loss through a combination of annealing and uniaxial drawing. By understanding the influence of the lamellar microstructure on amorphous relaxations that contribute to dielectric loss, the morphology can be tuned through appropriate processing conditions to minimize energy dissipation and improve capacitor performance. Chen et al. (2020) achieved flat-on primary crystals by nanoconfined crystallization in high-temperature PC/PVDF multilayer films (HTPC/PVDF MLFs) and studied its effect on dielectric insulation. Both flat-on primary and secondary PVDF crystals exhibited charge-blocking capability, thus capable of improving dielectric insulation for HTPC/PVDF MLFs. And it was observed that the MLFs with flat-on primary crystals had higher *η* and thus *U*_e_ than the melt-recrystallized MLFs with edge-on primary crystals. Therefore, MLFs with flat-on primary PVDF crystals should be more beneficial for dielectric insulation and energy storage.

**FIGURE 13:**
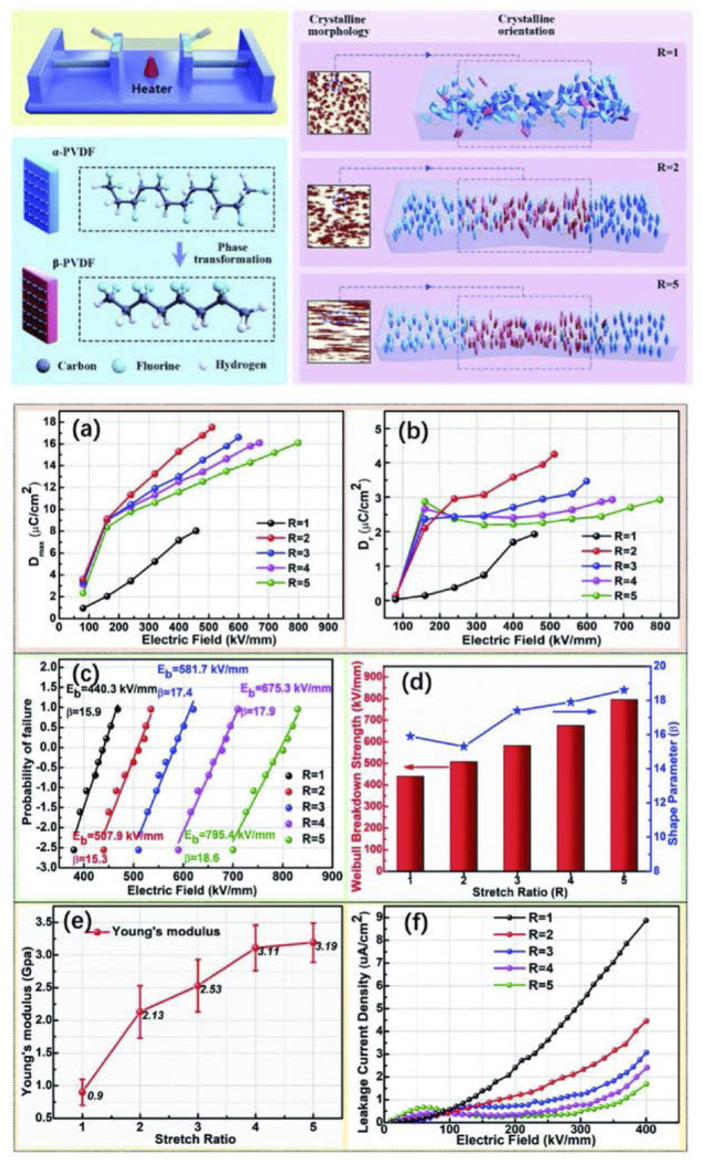
Schematic illustration of the uniaxial stretching process and structural development with increasing stretch ratio, and **(A)** The maximum electric displacement and **(B)** remnant electric displacement of the PVDF films as a function of the electric field; **(C)** Weibull distribution of electric breakdown strength; **(D)** The characteristic breakdown strength and shape parameters of the PVDF films with different stretch ratios; **(E)** Young’s moduli of the PVDF films with different stretch ratios; **(F)** Leakage current density of the PVDF films. Reproduced with permission from Ref. 111. Copyright 2021 The Royal Society of Chemistry.

Guo et al. (2021) fabricated a series of stretched PVDF polymer films demonstrated a substantial and concurrent increase in both electric displacement and breakdown strength by tuning its crystallization behavior in multi-aspects. The phase transition and crystal orientation during the mechanical stretching process caused a large increase in electric displacement, while the enhanced Young’s modulus and suppressed leakage current resulted in a significant improvement in the *E*_b_ (Figure 13). The promising results indicate the great potential of stretched PVDF films in energy storage and that they will be ideal candidates for next-generation power electronics and power-conditioning systems. Zhimin Dang’s group blended core-shell structured MBS rubber particles into P(VDF-HFP) polymer matrix to improve the intrinsic *E*_b_ and energy storage performances. Polarizing microscopy images show that blended films exhibit clear reduction of crystalline grains with the addition of MBS particles. The results show that an enhanced *E*_b_ of 515 MV/m and high *U*_e_ of 12.33 J/cm^3^ have been achieved in MBS-8 vol%/P(VDF-HFP) composite films attribute to the reduced crystalline grains, improved mechanical properties, volume resistance and suppressed leakage current of composite film by addition of MBS particles. These results provide a novel design of dielectric polymers for high *E*_b_ and *U*_e_ applications (Feng Q. K. et al., 2021).

Cui et al. (2020) prepared PMMA/PVDF all-organic films by coaxial spinning and hot pressing. Selection of the hot-pressing temperature can ensure that the PVDF maintains its original fiber shape while the PMMA is transformed into a continuous phase. A ferroconcrete-like structure is obtained that provides an increased amount of linear/ferroelectric micro-interfaces to increase the interface polarization. This structure endows the PMMA/PVDF all-organic films with a high *E*_b_ and an excellent *U*_e_. The results show that the structural design of all-organic films is controlled from a more microscopic perspective and new ideas are proposed for improving the performance of ferroelectric polymers.

So far, the effect of electrode-dielectric interface on the breakdown strength has received little attention, although metal-polymer interface plays a crucial role in controlling the dielectric performance in all flexible electronics. Pei et al. (2021) prepared an all-organic double-layer dielectric film consisting of PVDF as the matrix and PMMA as the organic nano-interlayer. The experimental results and computational simulations reveal that the surface morphology of dielectrics has a great effect on the electric field distribution at the electrode-dielectric interface, and further affects the leakage current and breakdown strength of the dielectric. These findings offer a new perspective to understand the impact of the electrode-dielectric interface on the polymer dielectric breakdown strength and this work provides a novel paradigm for fabricating polymer dielectrics with high *E*_b_ for energy storage.

Generally, in addition to the performance coupling relationship between the two dielectrics, the most important thing is the interface effect in multilayer dielectric. Since a large amount of interfacial polarization caused by interfaces may exist in multilayer systems, the study of such polarization characteristics is important for understanding the energy storage behavior and guiding the performance tuning of multilayer dielectrics.

### The other structure

**FIGURE 14:**
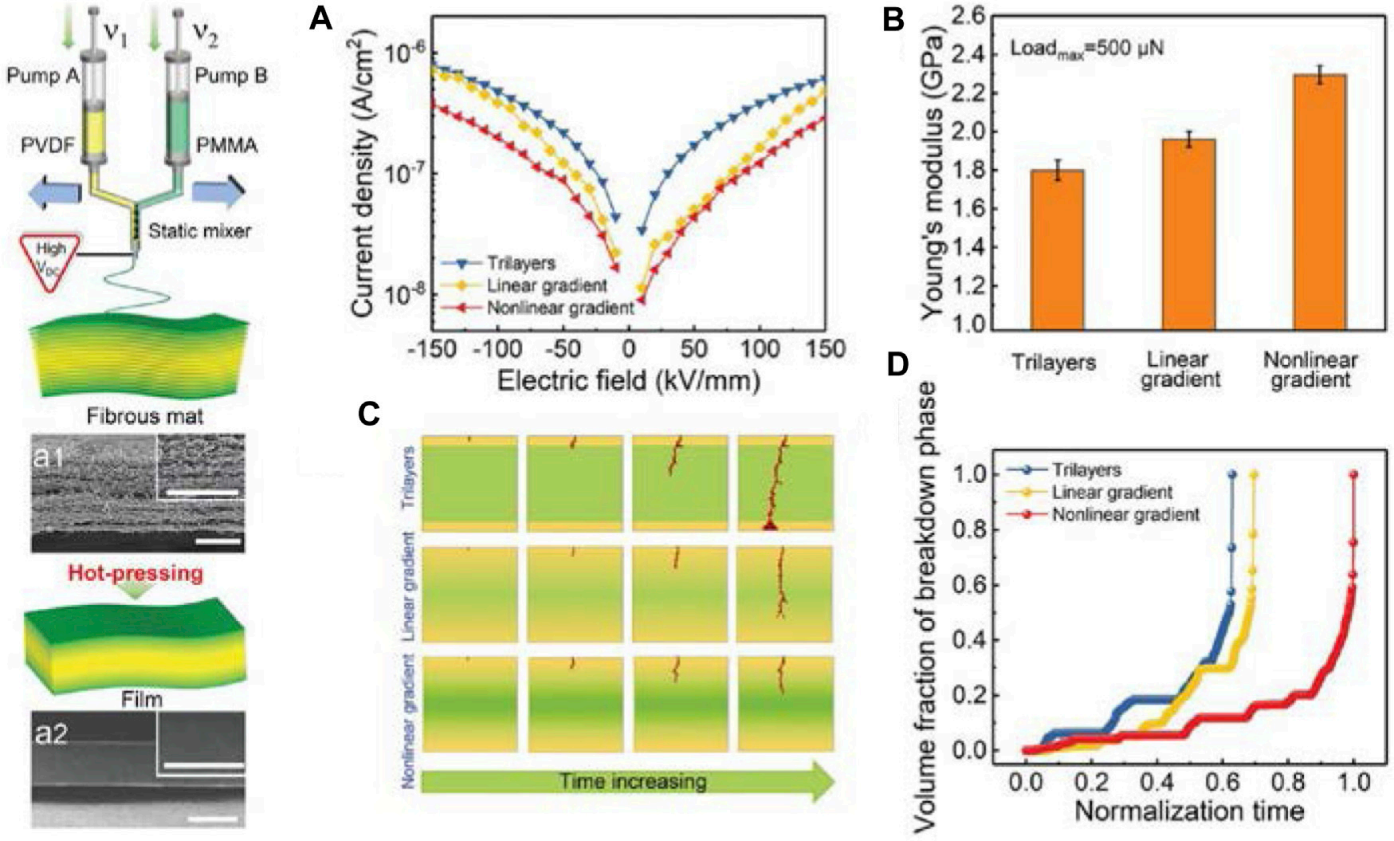
Schematic illustration of preparing all-organic PVDF/PMMA binary polymer film, and **(A)** The leakage current densities; **(B)** Young’s modulus for trilayer, linear gradient, and nonlinear gradient polymer films; **(C)** Simulated evolution of breakdown paths and **(D)** volume fraction of breakdown phase for trilayer, linear gradient, and nonlinear gradient polymer films. Reproduced with permission from Ref. 115. Copyright 2022 Wiley.

Besides the methods introduced above, there are other means of structural design that have been used to improve the energy storage performance of all-organic polymer dielectric materials. Jiang et al. (2022) proposed the continuously compositional gradient structure in PVDF-based all-organic dielectric polymers by modulating the spatial distributions of the PVDF/PMMA binary compositions, which leads to enhanced *D*_max_-*D*_r_ while simultaneously improved *E*_b_ compared to those of pure PVDF (Figure 14). The continuous out-of-plane composition gradient in films allows to tune electrical and mechanical behaviors, and thus induces a notably enhanced *E*_b_ by modulating the electromechanical breakdown process related to the coupling of local electric field and stress. As a result, an unprecedented high *U*_e_ of 38.8 J/cm^3^ and high *η* of 81% are achieved at the electric field of 800 kV/mm in the continuously gradient-structured dielectric polymer films. This work may provide a novel and simple way to develop high-performance polymeric dielectric materials to be used in high-energy-density dielectric capacitors for compact, efficient, and cost-reducing electronic and electrical systems.

Yuan M. et al. (2020) reported an all-organic composite comprising dielectric polymers blended with high-electron-affinity molecular semiconductors that exhibits concurrent high energy density and high *η* (90%) up to 200°C. They demonstrated that molecular semiconductors immobilize free electrons *via* strong electrostatic attraction and impede electric charge injection and transport in dielectric polymers, which leads to the substantial performance improvements. And this study offers a novel material strategy for tailoring the high-temperature capacitive performance of dielectric polymers. Chenyi (Zhou D. et al., 2022) have developed a series of novel polymer dielectrics, poly (arylene ether amide)s, with a giant high temperature energy storage density by designing the molecular structure. The results demonstrated that the polymer dielectrics with relatively great high-temperature capacitive properties exhibit a curly-packed structure at the molecular level. This work provides a new path to designing high-temperature polymer dielectrics by rationally constructing a curly-packed structure (Zhou Y.-N. et al., 2022).

It is well known that the properties of materials are determined by their structure. In addition to the microscopic molecular structure, the aggregated structure also has a significant impact on the properties of materials. Therefore, in the future, in addition to preparing polymer dielectric with novel chain structures by designing monomers, macroscopic structural design of existing polymers is also an effective way to prepare high *U*_e_ all-polymer dielectric.

## Summary and perspective

**FIGURE 15:**
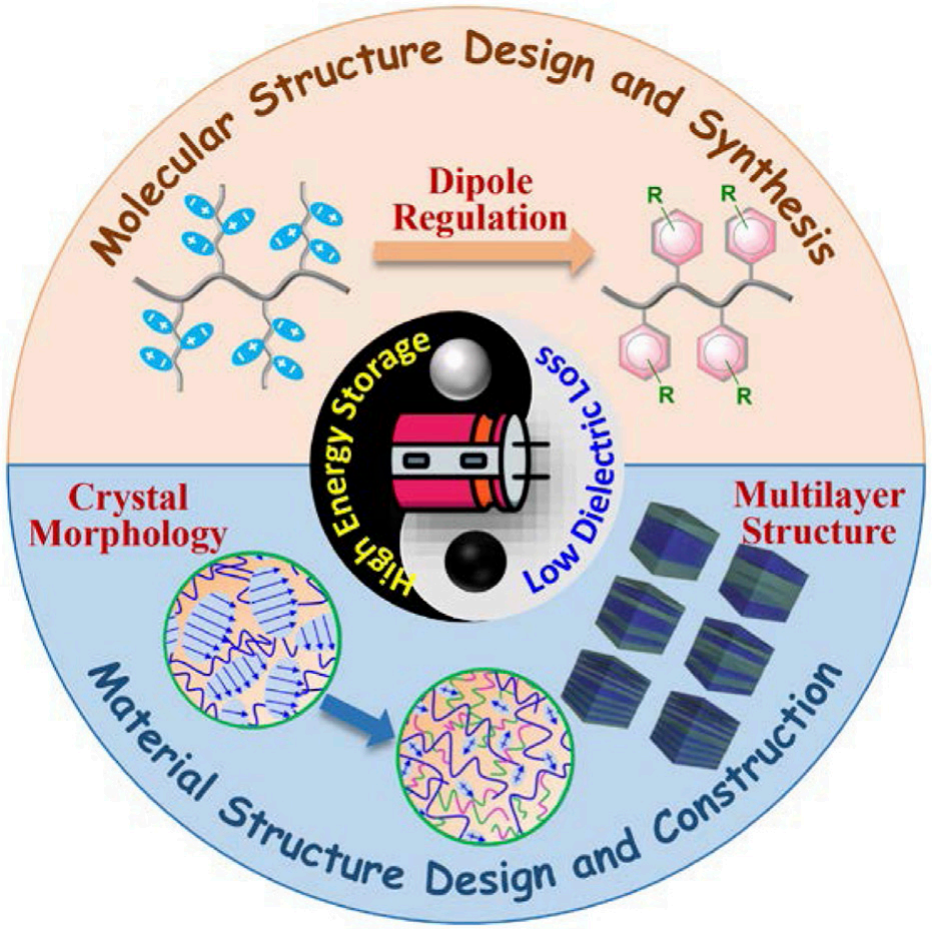
Schematics of remaining challenges and future prospects.

As an energy storage device, polymer-based film capacitors have received more and more attention with the rapid development of electromagnetic ejection, electric vehicles, electronic and electrical systems, renewable energy etc. owing to the unique advantages, such as high charge/discharge rate, high flexibility, and reliable self-healing capabilities. This review summarizes the basic theory of dielectrics, the application of CRP in polymer dielectric modification and synthesis, and the preparation of all-organic polymer dielectric composites with high energy storage properties through structural design. Although some important progresses have been made in all-organic polymer dielectric materials at home and abroad, there are still many challenges related to the field of all-organic dielectric polymers for film capacitors. In the future, the energy storage performance of polymer dielectrics can be regulated from both molecular structure and material structure, so as to achieve high energy storage density under the premise of low energy loss (Figure 15).

Firstly, the dielectric constant and the breakdown electric field of the dielectric is a pair of contradictions, they cannot be improved differently. Like PVDF-based ferroelectric polymers, although introducing defects by physical/chemical means can effectively reduce the size of the ferroelectric phase, thereby reducing the coupling effect between the ferroelectric phases and the resulting dielectric losses, but it is still far from the loss of BOPP. In response to this, we can start from the polymer molecular chain structure, and use a controllable method to design and synthesize new polymer dielectric materials with different topological structures, so as to solve the problems of high energy storage accompanying low breakdown electric field and high energy loss through means of dipole regulation.

Secondly, due to the high working temperature of potential dielectric devices, the requirements for polymer-based film capacitors, especially thermal management, are becoming more stringent, but the relatively low working temperatures of polymer dielectrics limit their application in next generation capacitors. Therefore, it is necessary to develop high temperature resistant all-organic polymer dielectrics. In the future, besides designing the molecular structure, the macrostructure of the material can also be controlled by using different process technologies, such as cross-linking, building a multi-layer structure, stretching, etc., so as to prepare high temperature resistant all-organic polymer dielectrics.

In addition, the dielectric film needs to be vapor-deposited to obtain a metallized film, which is then wound into a core, after that, a capacitor is obtained through series-parallel and encapsulation. Therefore, the processing procedure of film capacitors from dielectric films also should be paid more attention by industrial and academic communities. Different properties, including dielectric, thermal, mechanical, dispersion, and scattering characterizations, should be considered comprehensively during the manufacturing process of polymer-based film capacitors.

In conclusion, achieving large-scale, high-performance, and multifunctional all organic dielectric polymer materials is always a challenge. And this requires the efforts of multidisciplinary researchers including physicists, chemists, materials scientists, and electrical engineers.

## Glossary

ARGET-ATRP: Activators Regenerated by Electron Transfer for Atom Transfer Radical Polymerization
ATRP: Atom Transfer Radical Polymerization
BOPP: Biaxially Oriented Polypropylene
CRP: Controlled/living Radical Polymerization
*D*-*E*: Displacement-Electric Field Loop
*E*_b_: Breakdown Electric Field Strength
MBS: Methyl methacrylate-Butadiene-Styrene
MS: Poly(styrene-methyl methacrylate)
NMP: Nitroxide Mediated Polymerization
P(MMA-MAA): Poly(methyl methacrylate-methallyl alcohol)
P(St-MMA-MAA): Poly(styrene-methyl methacrylate-methallyl alcohol)
P(VDF-CTFE): Poly(vinylidene fluoride-chlorotrifluoroethylene)
P(VDF-HFP): Poly(vinylidene fluoride-hexafluoropropene)
P(VDF-TrFE-CTFE): Poly(vinylidene fluoride-trifluoroethylene-chlorotrifluoroethylene)
PBMA: Poly(butyl methacrylate)
PC: Polycarbonate
PEI: Polyetherimide
PEMA: Poly(ethyl methacrylate)
PET: Polyester
PMMA: Poly(methyl methacrylate)
PNBE: Polynorbornene
POFNB: Polyoxafluoronorbornene
PP: Polypropylene
PPS: Polyphenylene Sulfide
PS: Polystyrene
PVA: Poly(vinyl alcohol)
PVDF: Poly(vinylidene fluoride)
RAFT: Reversible Addition-Fragmentation Chain Transfer
ROMP: Ring-Opening Metathesis Polymerization
*T*_g_: Glass Transition Temperature
*U*: Charged Energy Density
*U*_e_: Releases Energy Density
*ε*_r_: Dielectric Constant
*η*: Releases Energy Efficiency
